# Accelerating acidic CO_2_ electroreduction: strategies beyond catalysts

**DOI:** 10.1039/d4sc04283b

**Published:** 2024-09-03

**Authors:** Bangwei Deng, Daming Sun, Xueyang Zhao, Lili Wang, Feiyu Ma, Yizhao Li, Fan Dong

**Affiliations:** a Huzhou Key Laboratory of Smart and Clean Energy, Yangtze Delta Region Institute (Huzhou), University of Electronic Science and Technology of China Huzhou 313001 China bwdeng@uestc.edu.cn yizhao@csj.uestc.edu.cn dongfan@uestc.edu.cn; b CMA Key Open Laboratory of Transforming Climate Resources to Economy Chongqing 401147 China; c School of Chemistry and Chemical Engineering, Lanzhou Jiaotong University Lanzhou 730070 China; d School of Environmental Science and Engineering, Southwest Jiaotong University Chengdu 611756 China

## Abstract

Carbon dioxide electrochemical reduction (CO_2_RR) into high-value-added chemicals offers an alternative pathway toward achieving carbon neutrality. However, in conventional neutral or alkaline electrolyte systems, a significant portion of CO_2_ is converted into (bi)carbonate due to the thermodynamically favorable acid–base neutralization reaction between CO_2_ and hydroxide ions. This results in the single-pass carbon efficiency (SPCE) being theoretically capped at 50%, presenting challenges for practical applications. Acidic CO_2_RR can completely circumvent the carbonate issue and theoretically achieve 100% SPCE, garnering substantial attention from researchers in recent years. Nevertheless, acidic CO_2_RR currently lags behind traditional neutral/alkaline systems in terms of product selectivity, stability, and energy efficiency, primarily because the abundance of H^+^ ions exacerbates the hydrogen evolution reaction (HER). Encouragingly, significant breakthroughs have been made to address these challenges, with numerous studies indicating that the regulation of the local catalytic environment may be more crucial than the catalyst itself. In this review, we will discuss the main challenges and latest strategies for acidic CO_2_RR, focusing on three key aspects beyond the catalyst: electrolyte regulation, local catalytic environment modification, and novel designs of gas diffusion electrodes (GDEs)/electrolyzers. We will also conclude the current advancement for acidic CO_2_RR and provide an outlook, with the hope that this technology will contribute to achieving carbon neutrality and advance towards practical application.

## Abstract 2

This review emphasizes the fundamentals of the CO_2_RR in neutral, alkaline, and acidic electrolytes and thoroughly discusses current challenges and strategies for acidic CO_2_RR beyond the catalyst itself.

## Graphical abstract

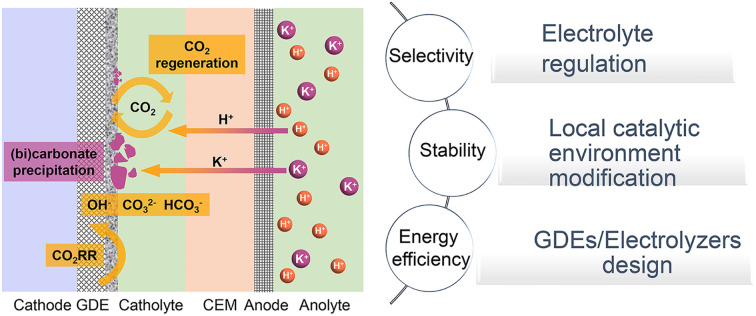

## 1. Introduction

The correlation between CO_2_ emissions and global warming has been extensively validated and recognized by the scientific community. The 28th Conference of the Parties to the United Nations Framework Convention on Climate Change (COP28), held in Dubai, UAE in 2023, highlighted that the current global efforts in various domains of climate action, including greenhouse gas reduction, are insufficient. To limit global temperature rise to within 1.5 °C, global greenhouse gas emissions must be reduced by 43% from 2019 levels by 2030.^1^ The CO_2_RR powered by renewable energy can significantly mitigate CO_2_ emissions while generating high-value chemicals, representing a viable negative carbon technology for achieving carbon neutrality.^2–9^ However, traditional CO_2_RR in neutral or alkaline electrolyte systems suffers from high carbon loss, resulting in low energy efficiency and challenges in scaling up.^10^ In contrast, acidic electrolytes, which contain a high concentration of H^+^, can effectively reduce or inhibit the formation of (bi)carbonates, allowing for *in situ* CO_2_ regeneration and addressing the issue of carbon loss.^11–13^ Consequently, acidic CO_2_RR has garnered considerable attention from researchers.

Current research on acidic CO_2_RR primarily focuses on catalyst development^14–21^ and the regulation of the local catalytic interface.^22–25^ However, although high carbon efficiency can be achieved in acidic CO_2_RR, it still faces significant technical and economic challenges for industrial applications. Firstly, since the reduction of H^+^ is more kinetically favorable than the CO_2_RR, especially in strong acidic electrolytes, the fierce competition from the HER poses a significant challenge to achieving high selectivity for C_2+_ products. Therefore, effectively suppressing the HER becomes a critical task for acidic CO_2_RR. Secondly, the addition of alkaline ions to inhibit the HER can also lead to (bi)carbonate precipitation locally, potentially decreasing the stability of the GDE, especially for membrane electrode assembly (MEA) electrolyzers. This is primarily due to the presence of a pH gradient near the electrode. As soon as the CO_2_RR commences, a locally elevated pH inevitably arises, leading to some of the CO_2_ to react with OH^−^ ions to form carbonate or bicarbonate. These species can further interact with alkali metal cations migrating from the anode, producing (bi)carbonates. If these (bi)carbonates are not fully dissolved in the electrolyte, the remaining portion will accumulate in the GDE or flow channel, thereby impacting the hydrophobicity and stability of the interface.^26^ To avoid (bi)carbonate formation in the presence of alkali cations, there seems to be a balance between the local and bulk pH. Notably, recent studies show that quaternary ammonium cations on the catalyst surface could replace the function of alkali cations, thereby efficient CO_2_RR can occur without metal cations.^27^ This method shows a promising ability to avoid the formation of (bi)carbonates and maintain long-term stability. Thirdly, it should be noted that most catalysts reported in the literature are still derived directly from neutral or alkaline systems, and since most metal oxides are not thermodynamically stable in acid and the active site might be destroyed, the stability of the catalyst in an acidic environment still needs to be enhanced.^18,19^ For example, since Koper *et al.*^28^ proved that CO_2_ electroreduction does not occur without metal cations, high concentrations of alkali metal cations in electrolytes are essential for acidic CO_2_RR in most reported studies. However, the simultaneous effect of anions (*e.g.*, Cl^−^ in KCl solution), which we discussed previously,^29^ can also significantly influence the structural evolution of catalysts and the performance of the CO_2_RR in acidic electrolytes, yet this aspect remains underexplored. Furthermore, Chen *et al.*^30^ reported a novel hollow-fiber GDE using only Cu metal as the catalyst, achieving a nearly 80% FE for C_2+_ products with a partial current density exceeding 2 A cm^−2^. The above research indicates that achieving practical industrial applications of acidic CO_2_RR requires more than just the development of a stable catalyst. Increasing evidence suggests that factors such as the electrolyte, GDE, and electrolyzer design are even more critical for achieving efficient acidic CO_2_RR.^31^ These aspects urgently require further in-depth investigation in the future.

**Fig. 1:**
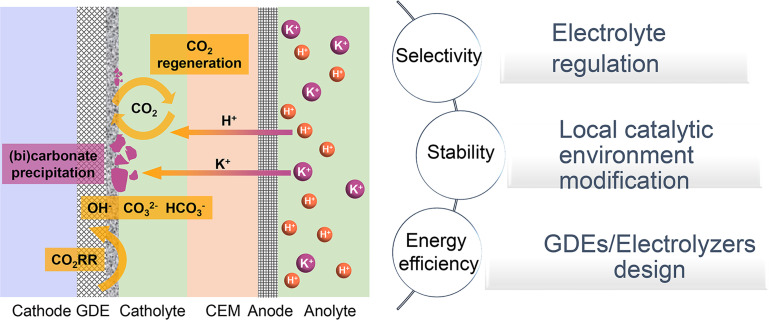
Schematic illustration of typical acidic CO_2_RR in a cation exchange membrane (CEM) based flow cell: current challenges and strategies.

In this review, we will investigate the mechanisms and primary strategies to enhance acidic CO_2_RR beyond the catalyst itself. We first summarize the state-of-the-art performance of the CO_2_RR under near-neutral, alkaline, and acidic electrolyte conditions, and then identify the main challenges of acidic CO_2_RR in three areas: selectivity, stability, and energy efficiency. Subsequently, we explore methods to address these challenges, including electrolyte regulation, local catalytic environment modification, and innovative GDE/electrolyzer designs (Fig. 1). Although existing review articles on acidic CO_2_RR provide valuable insights,^31–39^ our focus differs. For instance, Gu *et al.*^31^ discussed methods to improve the selectivity of acidic CO_2_RR *via* mass transport and electrode reactions, such as catalyst surface decoration, nanostructuring, and electronic structure modulation. Wang *et al.*^38^ addressed the carbonate issue primarily from the perspective of electrocatalysts for acidic CO_2_RR. Yan *et al.*^32^ focused on accelerating acidic CO_2_RR through the rational design of electrodes/catalysts and the local catalytic environment. Xia *et al.*^39^ explored the regulation of the reaction environment based on catalysts, electrodes, and electrolytes. Considering the primary challenges faced by acidic CO_2_RR from an industrial application viewpoint, we concentrate on factors beyond the catalyst itself, including the electrolyte, local environment, and GDE/electrolyzer. Our goal is to deepen the understanding of acidic CO_2_RR in terms of system design and process optimization, thereby promoting its industrial application in real-world environments.

## 2. Challenges of acidic CO_2_RR

**Fig. 2:**
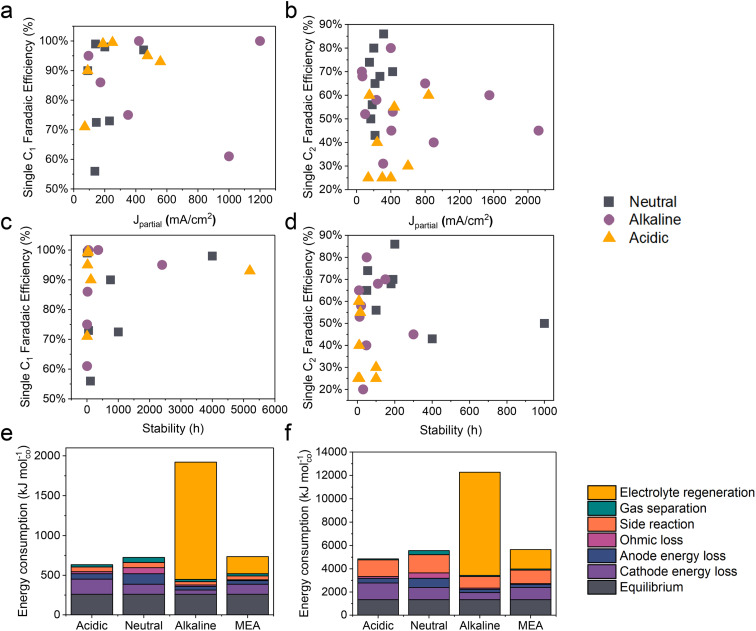
The state-of-the-art performance of the CO_2_RR in neutral, alkaline, and acidic electrolytes. The FE of single C_1_ and C_2+_ products *versus* (a and b) the corresponding partial current density and (c and d) the stability. Energy consumption (kJ mol^−1^) required to produce 1 mole of (e) carbon monoxide and (f) ethylene, assuming a partial current density of 200 mA cm^−2^. Systems based on a flow cell with acidic, near-neutral or alkaline medium, and an MEA with an anion exchange membrane, are compared.^12^

Compared to traditional neutral/alkaline CO_2_RR systems, the main advantages of acidic CO_2_ electroreduction are as follows: high carbon efficiency and high energy efficiency. By avoiding the formation of carbonates, which reduces CO_2_ loss, higher carbon efficiency can be achieved. Meanwhile, the ohmic losses in acidic electrolytes are lower than those in near-neutral electrolytes, which is crucial for achieving high energy efficiency at high current densities. However, the industrial application of acidic CO_2_ electroreduction still requires overcoming challenges such as poor product selectivity, inadequate system stability, and low energy efficiency (Fig. 2 and Tables 1 and 2).

**Table 1:** The state-of-the-art performance of the CO_2_RR (single C_1_ products) in neutral, alkaline, and acidic electrolytes

pH	Products	*J*_partial_ (mA cm^−2^)	FE (%)	Stability (h)	Voltage (V)	Electrolyte	SPCE (%)	Catalyst	References
Neutral	CO	200	98	4000	3	0.01 M KHCO_3_	—	Ag	40
		90	90	750	3.5	0.5 M KHCO_3_	—	Ag NW	41
		140	99	9	2.6	0.5 M KHCO_3_	—	Ni SACs@C	42
	HCOOH	145	73	1000	3.7	Pure water	—	Bi_2_O_3_	43
		450	97	—	−0.77 V RHE	1.0 M KHCO_3_	—	Grain boundary-enriched Bi	44
	CH_4_	230	73	50	4	0.1 M KHCO_3_	—	Cu NPs/N-doped carbon	45
		136	56	110	4.2	0.05 M KHCO_3_	—	Copper(ii) phthalocyanine	46
Alkaline	CO	1200	100	48	−1.2 V RHE	1 M KOH	—	Hg-CoTPP/N-doped graphene	47
		420	100	360	−1.2 V RHE	1 M KOH	—		
	CH_4_	350	75	5	−0.9 V RHE	1 M KOH	—	Cu(i)-based coordination polymer	48
		1000	61	5	−2 V RHE	1 M KOH	—	CuGaO_2_ nanosheet	49
	HCOOH	95	95	2400	−0.9 V RHE	1 M KOH	—	Sn–Bi/SnO_2_	50
		172	86	20	2.8	1 M KOH	—	Bi rhombic dodecahedra	51
Acidic	CO	475	95	20	3.55	0.5 M K_2_SO_4_ + H_2_SO_4_ (pH 0.5)	85	Ni–N–C	52
		250	100	36	−2.73 V RHE	1 M Cs_2_SO_4_ + H_2_SO_4_ (pH 2)	75.7	Ni–N–C	53
		188	99	25	−1.3 V RHE	0.1 M H_3_PO_4_ + 0.9 M KH_2_PO_4_ + 1.1 M KCl (pH 3)	64.3	Cu/Ni-NC	17
	HCOOH	558	93	5200	2.2	H_2_SO_4_ + 0.4 M K_2_SO_4_ (pH 1)	91	r-Pb	54
		90	90	125	−1.5 V RHE	0.05 M H_2_SO_4_ + 3 M KCl electrolyte (pH 1)	75	SiC-Nafion™/SnBi/PTFE	23
	CH_4_	71	71	5	3.6	0.005 M H_2_SO_4_	78	EDTA/CuPc/C NP	55

**Table 2:** The state-of-the-art performance of the CO_2_RR (single C_2_ products) in neutral, alkaline, and acidic electrolytes

pH	Products	*J*_partial_ (mA cm^−2^)	FE (%)	Stability (h)	Voltage (V)	Electrolyte	SPCE (%)	Catalyst	References
Neutral	C_2_H_4_	315	86	200	3	0.1 M KHCO_3_	—	Mesoporous Cu film on Cu foam	56
		200	80	—	3.2	0.5 M KHCO_3_	—	CuO nanoplates	57
		150	74	55	5	0.5 M KCl	—	CuO nanoplates	57
		272	68	180	3.6	0.1 M KHCO_3_	—	Quasi-graphitic C shell/Cu	58
		182	56	100	4.55	0.1 M KHCO_3_	—	Thiol/Ag–Cu	59
		420	70	190	3.3	1 M KHCO_3_	—	*N*-Aryl-substituted tetrahydrobipyridine/related oligomer	60
		166	50	1000	4.4	Pure water	39	Surface-step-rich Cu	61
		215	65	50	4.1	0.1 M KHCO_3_	—	Film/Cu	62
	EtOH	215	43	400	3.5	0.02 M KHCO_3_	—	Hydrophobic Cu dendrites	63
Alkaline	C_2_H_4_	400	80	50	1.5 V RHE	1 M KOH	—	Cu–Al	64
		800	65	9	−0.8 V RHE	1 M KOH	—	Antiswelling AEI-modified oxide-derived Cu nanosheets	65
		1550	60	—	−0.91 V RHE	7 M KOH	—	Cu-PFSA	66
		68	68	110	2.8	1 M KOH	18	PFSA ionomer coated Cu	67
		62	70	150	−0.55 V RHE	7 M KOH	—	Graphite/carbon NPs/Cu	68
	EtOH	100	52	—	−0.95 V RHE	1 M KOH	—	Defect-site-rich Cu	69
		406	45	300	−0.87 V RHE	1 M KOH	—	Nitrene surface functionalization Cu	70
		423	53	12	−0.75 V RHE	1 M KOH	—	F-Bonded, single K-doped Cu(111) nanocrystals	71
		232	58	20	−0.75 V RHE	1 M KOH	—	BaO/Cu	72
		900	40	48	3	1 M KOH	—	Cu_3_Sn	73
		2124	45	—	1.34	1 M KOH		Low-coordinated Cs modified Cu(200) nanocubes	74
		310	31	20	−0.75 V RHE	1 M KOH			
Acidic	C_2_H_4_	136	25	4.2	−1.41 V RHE	0.1 M H_2_SO_4_ + 0.4 M K_2_SO_4_ (pH 1.5)	—	Cu/C	12
		440	55	16	—	0.5 M H_3_PO_4_ + 0.5 M KH_2_PO_4_ + 2.5 M KCl (pH ∼1.7)	90	CoPc@HC/Cu	75
		150	60	10	—	0.05 M H_2_SO_4_ + 2.5 M KCl	70	EC-Cu	76
		400	25	100	5.5	0.05 M H_2_SO_4_ + 3 M KCl (pH 0.71)	51.8	Cu hollow fibers	30
		840	60	8	—	3 M KCl + HCl (pH 1)	25.49	Cu nanoneedles	21
		300	25	12	4.2	1 M H_3_PO_4_ +3 M KCl	77	Cation-augmenting layer-modified Cu nanoparticles	22
		240	40	10	−2 V RHE	1 M HCl + 1 M KCl (pH 1)	42	Cu gas diffusion electrode (GDL)	77
	EtOH	600	30	100	5.5	0.05 M H_2_SO_4_ + 3 M KCl (pH 0.71)	—	Cu hollow fibers	30

### 2.1 Selectivity

In acidic environments, the HER from H^+^ reduction typically dominates, making it challenging to selectively produce specific products, especially for high valued C_2+_ products such as ethylene, ethanol, *etc.* It can be observed from Fig. 2a and b that although the faradaic efficiency (FE) of C_1_ products under acidic conditions is comparable to that of neutral and alkaline conditions, its current density remains significantly lower than that of alkaline systems. Additionally, for C_2+_ products, both the current density and FE are lower compared to neutral and alkaline systems. These findings indicate that product selectivity under acidic conditions remains a focal research direction to overcome. Although current studies suggest that cation effects and local pH effects are critical in influencing the selectivity of the CO_2_RR in acidic environments, more fundamental or novel theoretical frameworks need further refinement.

### 2.2 Stability

The durability of electrodes and electrolyzers is critical to the industrial application of the CO_2_RR. It has been reported that the operating time of CO_2_ electrolysis cells should be comparable to that of hydrogen production electrolysis cells (over 50 000 h) and has a considerable decay rate.^78^ Specifically, the FE for single products (*e.g.*, CO, HCOOH, and C_2_H_4_) should be maximized (greater than 80%) and kept stable over extended periods (ΔFE/Δ*t*: <0.1% per 1000 h), while ensuring a low voltage decay rate (<10 μV h^−1^).^79^ At present, the longest stability for single C1 products in neutral, alkaline, and acidic electrolytes is 4000, 2400, and 5200 h respectively (Fig. 2c). It is exciting to note that Xia *et al.*^54^ recently reported a durable CO_2_ conversion in the proton-exchange membrane system. They utilized recyclable lead as the cathode catalyst, coupled with the hydrogen oxidation reaction (HOR), capable of running at over 5000 h at 600 mA cm^−2^ in acidic electrolytes while maintaining an FE_(HCOOH)_ of over 90% and a voltage of around 2.2 V. This study demonstrates the feasibility of achieving ultra-long stability in the CO_2_RR under acidic conditions. Apart from the intrinsic high stability of lead catalysts, the high stability of the GDE triple-phase interface, maintained by its surface hydrophobicity (crucially influenced by PTFE or carbon nanoparticles), plays a significant role. Moreover, by utilizing the HOR rather than the water oxidation reaction (WOR) at the anode, the overall voltage was decreased, and, more importantly, the generation of harmful hydrogen peroxide was avoided, which could degrade and even destroy the polymer electrolyte membrane (PEM).

However, in the case of C_2+_ products, the stability of all products is significantly lower than that of C1 products, with the highest stability currently below 1000 h (Fig. 2d). Additionally, in acidic systems, the FE and stability of C_2+_ products are also lower than in neutral or alkaline environments, indicating that the generation of C_2+_ products still poses a significant challenge in terms of stability. This phenomenon may primarily be attributed to the complex formation of C_2+_ products, which results in a more unstable three-phase interface. Factors such as local high pH-induced carbonation,^80^ electrowetting,^81^ liquid products crossing the membrane,^10^ and intermediate adsorption-induced catalyst reconstruction^8^ contribute to this instability. It is important to note that while acidic electrolytes can prevent carbonate precipitation and enhance CO_2_ conversion, current findings indicate that even in strongly acidic electrolytes (*e.g.*, pH = 1), local pH variations can still lead to carbonate precipitation. This not only increases CO_2_ reduction activity but also causes carbonate accumulation and affects product selectivity when the current density surpasses a certain threshold.^80^

### 2.3 Energy efficiency (EE)

Another key issue for the industrialization of the CO_2_RR is energy efficiency. Fig. 2e and f present an estimate of the energy required to produce 1 mole of CO and C_2_H_4_ based on a flow cell system using different media (acidic, near-neutral, and alkaline) and a proton exchange membrane MEA in CO_2_ electroreduction. The figure compares the energy consumption under each condition at a partial current density of 200 mA cm^−2^. In acidic medium, the electroreduction of carbon dioxide is more efficient due to the inhibition of the hydrogen evolution reaction by alkali metal ions, resulting in lower energy demand. However, the energy consumption in near-neutral and alkaline media is relatively high, possibly due to energy efficiency loss caused by carbonate formation. For instance, alkaline electrolytes such as KOH consume CO_2_, resulting in a carbon efficiency of less than 10% and the energy consumed for regeneration exceeds the electric energy consumed by the electrolyzer itself, which makes the CO_2_RR using alkaline electrolytes uneconomical.^12^

Note that due to the constant equilibrium potentials of CO_2_/CO and CO_2_/ethylene on the RHE scale, the overpotential increases with decreasing pH. Consequently, the energy consumption due to overpotential loss at the cathode follows the order: acidic > near-neutral > alkaline. Despite this, the overall energy consumption is lowest in acidic medium because of lower losses in other categories (Fig. 2e and f).^12^ In alkaline medium, the KOH electrolyte regeneration is the most energy-intensive, resulting in an overall energy consumption three times higher than in acidic medium. In near-neutral medium, ohmic and anode energy losses are higher than in acidic medium, leading to an overall energy consumption approximately 14% higher than in acidic medium.^12^

## 3. Electrolyte regulation

In previous discussions, we have explored the interfacial electrolyte effects on the CO_2_RR in neutral and alkaline environments, identifying local pH and alkali metal cation effects as the two most significant factors. When transitioning to an acidic electrolyte system, some mechanisms remain relevant, such as the persistence of local concentration gradients and the critical role of alkali metal cations. However, the proton concentration gradient in acidic systems introduces additional effects on reaction kinetics. In this section, we will examine how local pH and alkali metal cation effects influence the CO_2_RR under acidic conditions and discuss the future development prospects of cation-free acidic electrolyte systems.

### 3.1 Local pH effect

**Fig. 3:**
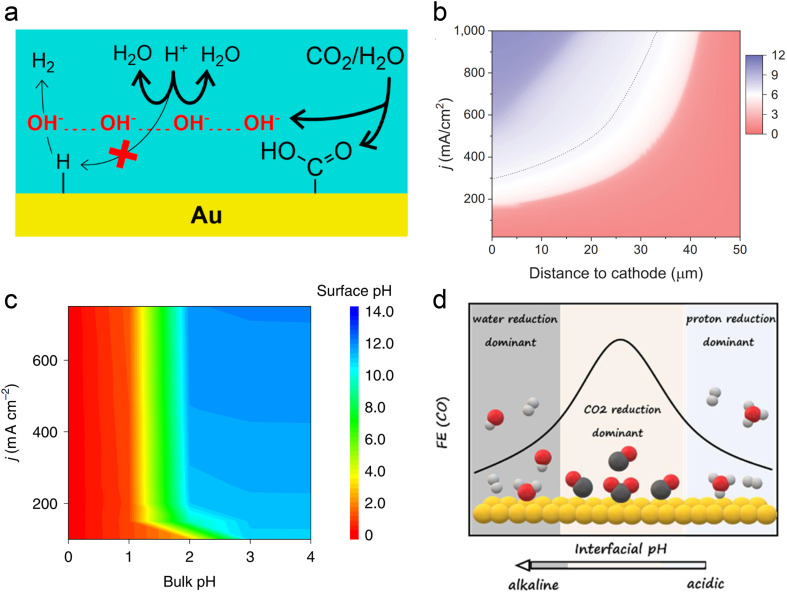
Local pH effect in acidic CO_2_RR. (a) Suppression of H^+^ reduction by OH^−^ generated from the CO_2_RR.^11^ (b) Modeling of pH at different distances to the cathode and current density in 1 M H_3_PO_4_ and 3 M KCl.^22^ (c) Surface pH at various applied current densities and bulk pH.^82^ (d) The dominant hydrogen evolution reaction (proton reduction *versus* water reduction) and CO_2_RR varied with interfacial pH from acidic to alkaline.^83^

In acidic systems, similar to traditional neutral or alkaline electrolyte systems, a local alkaline environment forms on the electrode surface during the CO_2_RR (Fig. 3a),^11^ leading to a gradient distribution of proton concentration (Fig. 3b).^22^ Previous studies have demonstrated that local pH primarily influences CO_2_ solubility and reaction kinetics.^29^ Generally, when the CO_2_RR causes an increase in the local OH^−^ concentration, H^+^ from the surrounding electrolyte diffuses to the surface to neutralize OH^−^. However, at higher current densities (*e.g.*, above 150 mA cm^−2^), the diffusion rate of H^+^ cannot keep up with the generation rate of OH^−^, leading to an increase in local pH. Consequently, a significant amount of CO_2_ forms (bi)carbonates with OH^−^ instead of undergoing the CO_2_RR. A recent *operando* synchrotron wide-angle X-ray scattering (WAXS) study demonstrated that substantial (bi)carbonate precipitation occurs in GDEs even in strongly acidic electrolyte (pH = 1).^80^ Nonetheless, if the proton concentration in the bulk phase is sufficiently high or proton diffusion is enhanced, the generated (bi)carbonates can react with H^+^ to re-form CO_2_ as a reactant. This is the key reason that acidic CO_2_RR can significantly increase the conversion rates. As can be seen, acidic CO_2_RR requires a trade-off between local and bulk pH, where the local pH remains relatively alkaline to promote the CO_2_RR while inhibiting the HER,^11^ without causing excessive accumulation of (bi)carbonates, and thereby guaranteeing sufficient CO_2_ at the reaction interface. Indeed, Wang *et al.*^82^ modelled local pH changes in the vicinity of the electrode surface at varied current density with different bulk pH (Fig. 3c). They proposed that bulk pH 2 might be available to balance high local CO_2_ and high current density without significant carbonate formation.

In fact, Koper *et al.* initiated the study of acidic CO_2_RR in 2015, exploring the relationship between proton dependence and the selectivity of various products.^84^ From a thermodynamic perspective, the HER can proceed *via* proton reduction or water reduction, as any Brønsted acid can act as a proton donor.^29,85^ However, Koper *et al.* posited that under typical CO_2_ reduction conditions, water reduction remains the predominant form of the HER. This conclusion is primarily based on their observation that the onset potential for water reduction is significantly influenced by the CO_2_ reaction, whereas the onset potential for proton reduction remains relatively unaffected under the same conditions, even in an acidic electrolyte (pH = 2.5).^86^ On the other hand, when proton transport kinetics are considered, such as at lower pH levels, H^+^ reduction also becomes a dominant pathway for the HER.^31^ Koper *et al.*^83^ recently investigated acidic CO_2_RR on planar Au electrodes, detecting the changes of FE varied with interfacial pH. They demonstrated that the primary HER shifts from proton reduction to water reduction as the local environment transitions from acidic to basic. Meanwhile, the CO_2_RR initiates in the proton reduction region and dominates under the near-neutral conditions. Since protons are consumed by OH^−^ generated from CO_2_ reduction; proton reduction diminishes while the CO_2_RR persists (Fig. 3d). Overall, the above theory suggests that the HER in acidic CO_2_RR can be suppressed by either increasing the local pH or limiting the concentration of interfacial water molecules.

To increase the local pH and promote the acidic CO_2_RR, Gu *et al.* posited that the key is to limit the migration of protons rather than to retard the kinetics of H^+^ reduction, because the onset potential of H^+^ reduction is more positive than that of the CO_2_RR and it always reaches the mass-transport limit under CO_2_ reduction conditions.^31,82^ They proposed three different ways to regulation the mass transport process, which include creating high local pH by the CO_2_RR, suppressing diffusion of H^+^ and migration of H^+^ by alkali cations (the cation effect will be discussed in Section 3.2).^31^ More recently, they demonstrated that the key role of the Ni–N–C catalyst in acidic tandem CO_2_RR-to-C_2+_ is as a local pH modulator, instead of solely producing the CO intermediate for Cu. The catalyst-induced high local pH is the major reason for the improved C_2+_ formation under acidic conditions.^87^ However, to further improve the selectivity of single C_2+_ products, *e.g.*, ethanol, the adsorption and coverage of *CO cannot be ignored, based on a recent study which showed that the presence of hydronium ions (H_3_O^+^) in acidic electrolyte might weaken the *CO binding energy and induce a low coverage on Cu.^88^

Given the above discussion, the direct measurement of local pH becomes vital for the deep understanding of pH effects. Several methods have been reported, for example, the Raman or IR peak area ratio of HCO_3_^−^ (1014 cm^−1^) to CO_3_^2−^ (1067 cm^−1^) is linearly correlated to the surface pH in the vicinity of the electrode,^89,90^ according to the Henderson–Hasselbach equation.^91^ Moreover, Zhong *et al.*^92^ reported that *para*-mercaptobenzoic acid (*p*-MBA) can be used as a nanoscale pH meter to monitor the local pH near the electrode surface during the electrochemical reactions. At low pH, the carboxylate group in *p*-MBA was mostly protonated, and at high pH values, the carboxylate group was mostly deprotonated. These changes in molecular structure were reflected in the Raman spectra, specifically the bands at 1393 cm^−1^ (COO^−^) and 1702 cm^−1^ (C=O). Thus, the pH value can be obtained from the intensity ratio of the two peaks: (COO^−^)/(C=O). The (COO^−^)/(C=O) ratio at pH 4–10 showed good reproducibility, while it varied largely at pH < 4 or pH > 10.

In addition, apart from spectroscopic methods, C. Co *et al.*^93^ proposed that the rotating ring-disc electrode (RRDE) is a versatile tool for detecting local pH change at various catalyst surfaces, where the local pH change can be measured by the CO (as the probe molecule) oxidation peak potential shift that varied with the local concentration of OH^−^. Another way to evaluate the surface pH is based on the redox potential change (*e.g.*, the oxidation of Cu^0^ to Cu^+^), since the redox potentials are pH independent on an RHE scale; however, when the local pH (pH_surface_) is different from the bulk (pH_bulk_), the onset oxidation potential of Cu can change. Finally, the pH_surface_ can be calculated by using the following equation:^77^*E* (*vs.* RHE) = *E* (*vs.* Ag/AgCl) + 0.209 V + 0.0592 × pH_surface_pH_surface_ = pH_bulk_ + ΔpH

### 3.2 Alkali cation effect

**Fig. 4:**
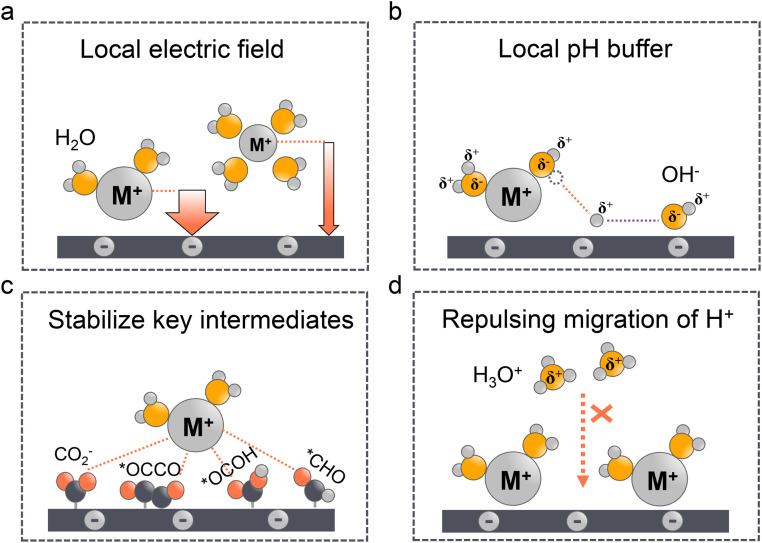
Alkali cations effect in acidic CO_2_RR. (a) Local electric field: larger cations are less hydrated and prefer to absorb in the outer Helmholtz plane (OHP). (b) Local pH buffer: the hydrated cations with lower p*K*_a_ can give more protons to neutralize the locally generated OH^−^. (c) Stabilize key intermediates: the hydrated cations could stabilize the key intermediates (*e.g.*, CO_2_^−^) effectively *via* electrostatic interactions. (d) Repulsing migration of H^+^: the accumulated cations in the OHP can repulse the migration of H^+^ and affect the local pH.

Since local pH is proved vital for acidic CO_2_RR, as a matter of fact, once it reaches a local alkaline environment, the concentration and type of alkali metal cations become the key factors to determine the selectivity of C_2+_ formation. In fact, the cation effect in neutral or alkaline electrolytes has been thoroughly discussed in our^29^ and other works.^32,94–96^ Three main theories have been proposed including the modification of the local electric field (Fig. 4a), the local pH buffer (Fig. 4b), and the stabilization of key intermediates (Fig. 4c). We will not delve into the specific analysis of the three aforementioned mechanisms in this study in detail. Instead, our focus will be on examining the applicability of these theories in acidic electrolyte systems and their crucial role in inhibiting the migration of H^+^ ions (Fig. 4d).

The larger cation effect was first investigated by Frumkin^97^ in 1959; the larger cations could increase the overall current density, which they attributed to higher specific adsorption of larger cations and the increased potential in OHP. However, this theory cannot explain the selectivity of the CO_2_RR over the HER. Moreover, since the equilibrium adsorption potential of alkaline cations (*e.g.*, K^+^) is more negative than that of the CO_2_RR, their specific adsorption might not occur under these conditions.^98^ Markovic *et al.*^99^ ruled out the specific adsorption theory and proposed that the accumulation of cations near the surface was due to the noncovalent interactions, which finally result in the high local electric fields. Ringe *et al.*^100^ and Resasco *et al.*^101^ then proved that larger cations are less likely hydrated and prefer to adsorb in the OHP, which can increase the surface charge density and the corresponding electronic field (Fig. 4a). Meanwhile, in acidic electrolyte, the cation-induced local electric field increases the CO_2_ activation kinetics such that it is more pronounced than that in the neutral or alkaline electrolytes. Sargent *et al.*^22^ showed that the Tafel slope decreased with the increase of K^+^ concentration in H_3_PO_4_ electrolytes. And more importantly, the Tafel slope exhibited an extremely high value in the absence of K^+^, indicating the key role of cations in facilitating the kinetics of the CO_2_RR.^28^ Several studies have also proved that the high concentration of larger alkaline cations will promote the CO_2_RR over the HER in acidic electrolytes.^102–104^ However, these conclusions are always reached in flow cells, which may not be directly used in MEA electrolyzers. Due to the absence of electrolyte in the cathode electrode, the higher concentration of alkaline cations could also promote (bi)carbonate precipitation in GDEs, thereby affecting the long-term stability.^10^ Pan *et al.*^105^ recently demonstrated that an optimal concentration of H^+^ and Cs^+^ in acidic MEA must be maintained to balance carbonate deposition and CO_2_RR performance. A recent work from Bao's group^52^ also showed that the coexistence of H^+^ and K^+^ can synergistically stabilize the *CO_2_ intermediate and promote the formation of CO.

The pH buffer effect of cations was initially proposed by Singh *et al.*^106^ in 2016. In contrast to the local electric field effect mentioned above, they demonstrated that under negative potentials, larger cations could induce stronger electric fields. Consequently, the p*K*_a_ value of hydrated cations decreases, allowing them to function as a pH buffer by providing more protons to neutralize the locally generated OH^−^. This ability to tailor the local pH prevents it from becoming excessively high, which could reduce the reactive CO_2_ concentrations (Fig. 4b). The pH buffer effect suggests that maintaining a stable and balanced local pH may be more advantageous for the CO_2_RR. This effect is particularly pronounced in acidic electrolytes, where a higher local pH can effectively suppress the HER, but excessively high pH levels may result in CO_2_ loss, undermining the benefits of high CO_2_ conversion rates in acidic systems. Yan *et al.*^32^ have also touched upon this concept in a recent review, although further research is needed to elucidate the specific mechanism of the pH buffer effect in acidic environments. On the other hand, it is worth noting that previous studies have indicated that the pH buffer effect may not always be reliable. For instance, by using *in situ* surface-enhanced infrared absorption spectroscopy (SEIRAS), Ayemoba *et al.*^107^ suggested that while the pH buffer effect may exist, its magnitude could be overestimated.

The third theory of stabilizing the key intermediates of the CO_2_RR was first proposed by Chen *et al.* and further developed by Resasco *et al.* (Fig. 4c). Particularly, Koper *et al.*^28^ later precluded the effect of local electronic field and pH buffer on hydrated cations, because they found that the CO_2_RR does not occur in the absence of metal cations in the solution. Since the local electronic field and pH buffer effect should only affect the reaction kinetics the CO_2_RR should still take place in cation-free electrolytes. This finding supports the theory that the hydrated cations could stabilize the key intermediates (*e.g.*, CO_2_^−^, *OCCO, *OCOH, and *CHO) effectively *via* electrostatic interactions. Indeed, Huang *et al.*^108^ reported that K^+^ could stabilize the *OCOH intermediate and promote HCOOH formation in strong acid media (pH = 1). More recently, Sun *et al.*^109^ demonstrated that the regulation of the CO_2_RR in acid by cation effects also involves alterations in the water structure. Specifically, Li^+^ effectively promotes the adsorption of CO_2_ but slows down the hydrogenation rate, while larger cations such as Na^+^ accelerate the CO_2_RR through a more flexible water network. This study indicates that cations can influence the adsorption and activation of CO_2_ by modulating the interfacial water structure.

**Fig. 5:**
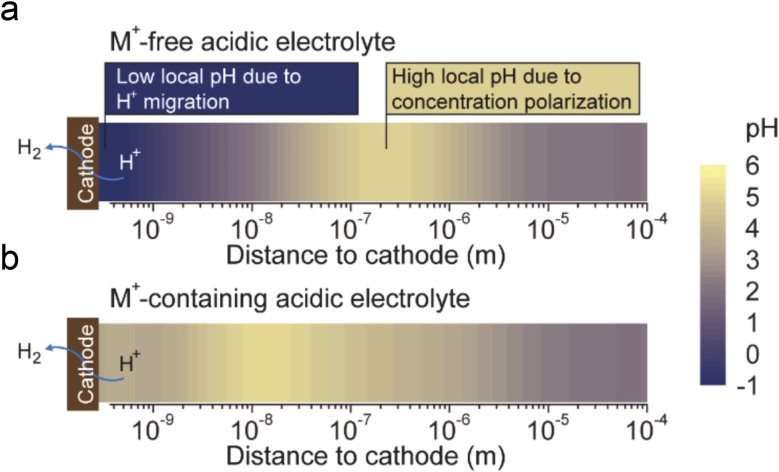
The pH distribution values near the cathode under conditions where H^+^ reduction reaches the plateau current density (limiting mass transport condition):^31^ (a) in acidic electrolyte free of alkali cations, (b) in acidic electrolyte containing alkali cations.

The fourth theory, repulsing the migration of H^+^, was recently proposed in acidic CO_2_RR (Fig. 4d). Since the biggest obstacle is the suppression of the HER, Gu *et al.*^110^ investigated the role of alkali cations in suppressing the reduction rates of H^+^ in acidic electrolytes. They showed that when the concentration of cations (*e.g.*, Li^+^) is higher than that of H^+^ in the bulk electrolyte, the migration of H^+^ could be completely suppressed. Since the H^+^ ions are continuously consumed *via* the HER near the electrode surface, while the alkali cations are chemically inert, the accumulation of alkali cations dominated in the OHP than H^+^.^31^ As a result, the suppression of H^+^ migration could also significantly affect the local pH near the surface. Recent simulation results show that once the H^+^ reduction reaches the limiting mass-transport condition (plateau current density), when approaching the cathode surface, H^+^ ions are firstly consumed near the cathode, leading to a reduction in H^+^ and a consequent increase in local pH. Subsequently, due to electrostatic attraction, hydrogen ions migrate back towards the cathode, causing a decrease in local pH. The maximum local pH is observed approximately 100 nanometers from the cathode, while the pH at the cathode surface remains significantly lower than the bulk pH (Fig. 5a). However, when alkali metal ions are present, the maximum local pH value appears approximately 10 nanometers away from the cathode, with the pH on the cathode surface being higher than the bulk electrolyte pH (Fig. 5b). Notably, the local pH of the cathode's OHP increases when the cathode potential shifts negatively, but this occurs only in acidic solutions with alkali metal ions. In contrast, in acidic solutions without alkali metal ions, regardless of the overpotential, continuous H^+^ migration replenishes H^+^ at the cathode surface, maintaining the local pH at the cathode OHP at −0.9.

### 3.3 Cation-free electrolyte

Recent studies, including the above observations, indicate that alkali cations are indispensable in acidic CO_2_RR. However, the primary advantage of acidic electrolysis is to avoid the formation of (bi)carbonate and enhance carbon efficiency. The presence of alkali cations introduces two main challenges to the sustainability of the CO_2_RR. The first issue is the formation of (bi)carbonate precipitates due to local pH increase and subsequent electrostatic attraction with accumulated alkali cations. These hydrophilic precipitates disrupt the hydrophobicity of the GDE, leading to cathode flooding. While reducing the concentration of alkali cations can mitigate this issue, it also compromises the selectivity of the CO_2_RR. The second issue is pH variation in both the catholyte and anolyte. As the CO_2_RR progresses, the pH of the catholyte approaches neutral or alkaline, while the anolyte becomes more acidic. This variation poses significant challenges for the long-term electrolysis of acidic CO_2_RR.^31^

**Fig. 6:**
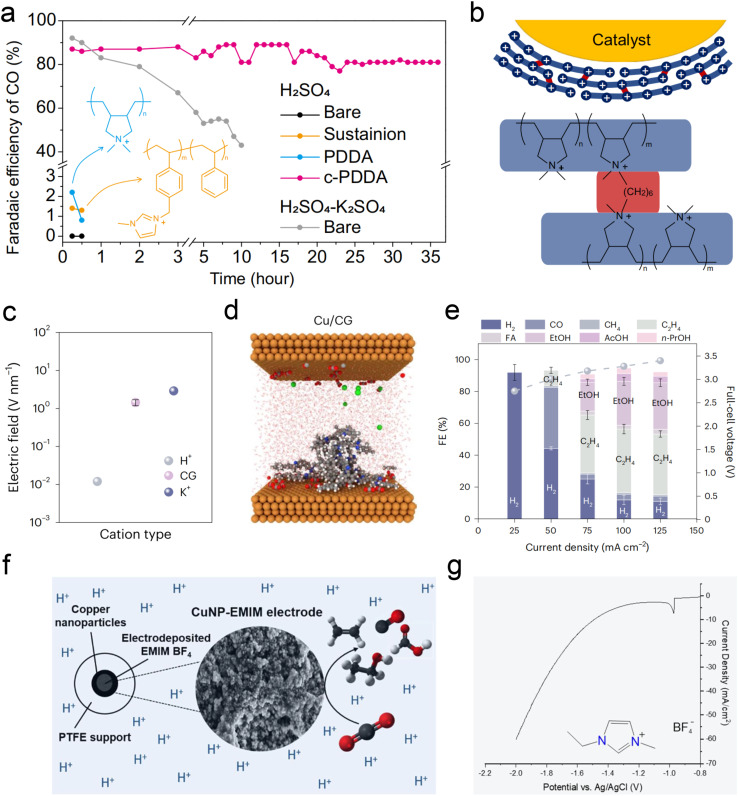
Current strategies for acidic CO_2_RR in metal cation-free electrolytes. (a and b) FE of CO during electrolysis with a constant current density of 200 mA cm^−2^. Catalysts of bare Ag, Sustainion-, PDDA-, and c-PDDA-decorated Ag with 0.1 M H_2_SO_4_ or 0.1 M H_2_SO_4_ + 0.4 M K_2_SO_4_ as flowing electrolyte.^25^ (c and d) Computational studies of electric field for cationic groups (CG)-functionalized catalysts in acidic CO_2_RR, comparison of H^+^, K^+^, and immobilized CG at OHP. Note that the interfacial electric field generated by CG was of the same order of magnitude as that generated by K^+^.^111^ (e) Full-cell-voltage performance of carbon-protected CG-medium Cu in a slim flow cell, at applied current densities from 25 mA cm^−2^ to 125 mA cm^−2^ with 0.2 M H_2_SO_4_ flowing electrolyte.^111^ (f) The electrodeposition of an imidazolium-based layer on Cu NPs enables acidic CO_2_RR in the absence of alkali cations.^112^ (g) The corresponding LSV curves of electrodeposition in 0.5 M EMIMBF_4_ aqueous solution using a Cu NP GDE cathode.^112^

Fortunately, recent studies have found some ways *via* using metal cation-free electrolytes to solve the above problem. The critical role of alkali metal cations in acidic CO_2_RR lies in their accumulation on the cathode surface, which subsequently alters the local electric field, proton concentration, and reaction kinetics. The local electric field effect of these cations is pivotal for the stability of key intermediates. In light of this, Gu *et al.*^25^ initially explored the possibility of fixing cations on the catalyst surface as an alternative to introducing cations in the bulk electrolyte. They proposed using the high-density quaternary ammonium cations in poly-dimethyl-diallyl-ammonium chloride (PDDA) to mimic the local electric field effect of alkali metal cations (Fig. 6a). Due to the water solubility of PDDA, to prevent it from being washed away by the electrolyte during the reaction, it was cross-linked (c-PDDA) and immobilized on the catalyst surface (Fig. 6b). This approach ultimately demonstrated excellent CO generation selectivity and stability in pure H_2_SO_4_ electrolyte. The bulk pH of the catholyte and anolyte is also relatively stable during 10 h electrolysis in pure H_2_SO_4_ electrolyte. Similarly, Li *et al.*^113^ fixed the PDDA on graphene oxide (GO) *via* electrostatic interactions, they achieved an FE of 85% and carbon efficiency of 93% for CO formation in pure H_2_SO_4_. More importantly, they also conducted the test in pure water electrolyte, and obtained 78% FE for CO formation at 100 mA cm^−2^.

Sinton *et al.*^111^ sprayed Aemion^+^ ionomer, containing quaternary ammonium cations, onto the surface of Cu/PTFE, resulting in a surface electric field strength equivalent to that of alkali metal ions (Fig. 6c and d). This treatment achieved an 80% C_2+_ FE at a current density of 100 mA cm^−2^ in pure H_2_SO_4_ (pH = 0.4) electrolyte (Fig. 6e). Additionally, Fontecave *et al.*^112^ deposited 1-ethyl-3-methylimidazolium tetrafluoroborate (EMIMBF_4_) ionic liquid, containing imidazolium cations, onto the Cu surface to simulate the alkali metal K^+^ ion layer. The movement of H^+^ and the HER can also be suppressed effectively (Fig. 6f and g). Interestingly, Zhu *et al.*^114^ recently discovered that in pure acidic electrolyte (pH = 1), even without the modification of the aforementioned organic cation layer, the Co–N site in cobalt phthalocyanine can effectively stabilize the *CO_2_ intermediate and generate CO with an FE of 60%, even though the selectivity still needs to be improved.

## 4 Local catalytic environment modification

**Fig. 7:**
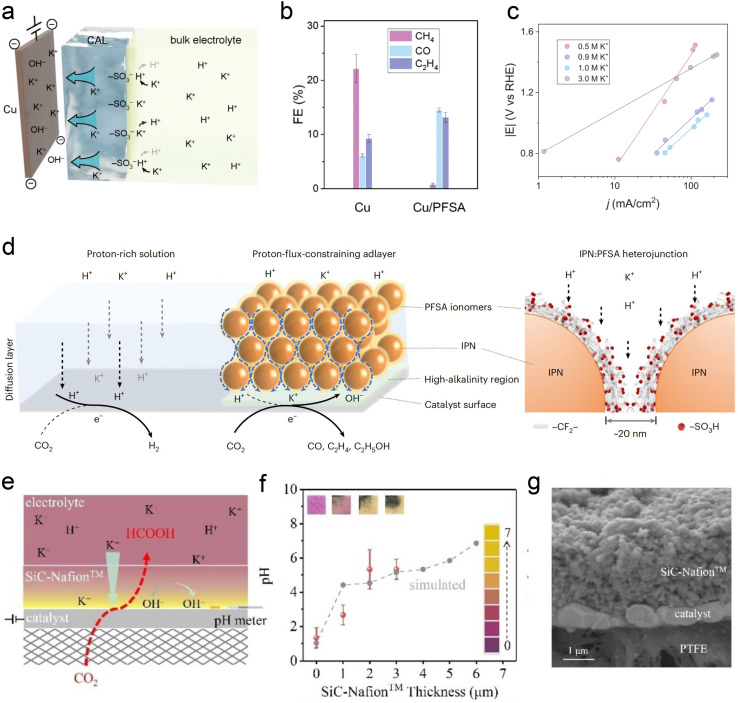
Ionomer-modified local catalytic environment. (a) Schematic illustration of ionic environment and transport near the catalyst surface functionalized by the PFSA ionomer.^22^ (b) FEs toward gaseous CO_2_RR products on bare Cu and PFSA-modified Cu (Cu/PFSA) at 400 mA cm^−2^ in 1 M H_3_PO_4_ with 3 M KCl.^22^ (c) Tafel slopes obtained in electrolyte with different K^+^ concentrations.^22^ (d) Schematics of interfacial reactions and proton transport near the catalyst surface *via* proton-flux-constraining ionomer adlayer design.^24^ (e) Schematic of catalysts during the CO_2_RR at pH 1.^23^ (f) Surface pH *vs.* SiC-Nafion™ layer thickness. (g) Cross-section SEM image of SiC-Nafion™/SnBi/PTFE.^23^

Similar to the intrinsic structure of the catalyst, the local catalytic reaction interface is a critical factor influencing the performance of the CO_2_RR. To address the current challenges faced by acidic CO_2_RR, such as selectivity and stability, recent research primarily focuses on the surface modification of the catalyst and the regulation of the carrier. Sargent *et al.*^22^ proposed using cationic perfluorosulfonic acid (PFSA) ionomer (*e.g.*, Nafion) to modify the Cu surface. Its acidic –SO_3_H group is expected to exchange its protons with K^+^ from the bulk electrolyte in a nonacidic local environment, maintaining a high K^+^ concentration at the catalyst surface (Fig. 7a). The selectivity of C_2+_ products was significantly enhanced over C_1_ products, and they finally achieved the CO_2_RR on Cu at pH < 1 with a single-pass CO_2_ utilization of 77%, including a conversion efficiency of 50% toward C_2+_ products (Fig. 7b). Moreover, their work demonstrated that the Tafel slope decreases with the increase of alkali cation concentration, leading to faster kinetics of acidic CO_2_RR. This result further proves the key role of accumulated alkali cations in the vicinity of the cathode electrode (Fig. 7c). Similarly, Sinton *et al.*^24^ reported a COF:PFSA-adlayer-modified Cu electrode, creating evenly distributed cation-carrying and hydrophilic–hydrophobic nanochannels that control the catalyst microenvironment (Fig. 7d). The modifier acted as a proton-flux-constraining ionomer adlayer. The resulting high local alkalinity and cation-enriched environment enables a C_2+_ FE of 75% at 200 mA cm^−2^ in a strongly acidic electrolyte (pH = 1). This PFSA-modifier also shows a similar function in acidic tandem CO_2_RR.^76^ Zhong *et al.*^23^ recently reported an electrically nonconductive nanoporous SiC-Nafion™ layer, which can maintain near-neutral conditions on the surface (Fig. 7e and f).The SnBi catalyst can also be stabilized without corrosion, and obtain an FE_(HCOOH)_ of >90% at 100 mA cm^−2^ over 125 h. Zhao *et al.*^13^ used quaternary ammonium poly(*N*-methyl-piperidine-*co-p*-ter-phenyl) (QAPPT) and PTFE to co-modify a commercial Ag catalyst, utilizing the electrostatic repulsion of quaternary ammonium salts to reduce the diffusion of H^+^ and K^+^ and promote the selectivity of acidic CO_2_RR in an MEA reactor. It should be noted that most of the above modifications are conducted in flow cells, and high concentrations of alkali cations (*e.g.*, KCl) are always indispensable.

**Fig. 8:**
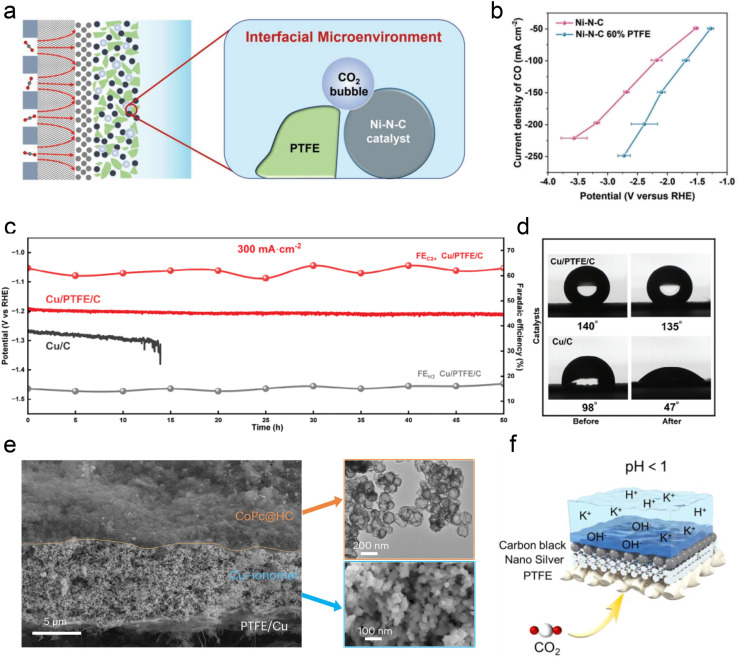
The modification of the local catalytic environment with other substances, such as PTFE, polymers, and carbon supports. (a) Schematic illustration of the interface microenvironment inside the catalyst layer with added PTFE. An established balance between gaseous CO_2_ and liquid electrolyte in the catalytic layer of GDEs.^53^ (b) CO partial current density of Ni–N–C and Ni–N–C 60% PTFE electrodes at various current densities.^53^ (c) Cathode potential and FE for C_2+_ and H_2_ on the Cu/C and Cu/PTFE/C electrodes in an additional 50 h durability test at a current density of 300 mA cm^−2^.^115^ (d) Cu/C and Cu/C/PTFE electrodes before and after the CO_2_RR at −1.2 V *vs.* RHE.^115^ (e) Cross-sectional SEM image of a CoPc@HC/Cu tandem electrode (left). TEM image of the upper CoPc@HC catalyst layer and SEM image of the lower Cu catalyst layer with the Cu–ionomer interface (right).^75^ (f) Schematic illustration of the carbon black layer that promotes the accumulation of K^+^ at pH < 1.^116^

Besides ionomer modifiers, other substances such as PTFE, various polymers, and carbon supports have been utilized to optimize the local catalytic environment. Interestingly, Li *et al.*^53^ demonstrated that incorporating hydrophobic and non-conductive PTFE into a Ni-SAC catalyst reduces system impedance. The presence of PTFE is believed to regulate the balance between CO_2_ and H_2_O, thereby decreasing the thickness of the interfacial diffusion layer and enhancing the selectivity and stability of acidic CO_2_RR (Fig. 8a and b). Lin *et al.*^115^ recently showed that the intrinsic superhydrophobicity of the microporous layer (MPL) in a GDE is influenced by ionomers, such as Nafion, present in the catalyst slurry. Hence, by introducing PTFE suspension and adjusting the electrodeposition time, the hydrophobicity of the catalyst surface can be effectively preserved, thereby enhancing the stability (Fig. 8c and d). In addition, Sargent *et al.*^76^ introduced amide-bearing polymers (*e.g.*, poly(Lys, Phe)) during the electrodeposition of Cu catalysts, which increases the coverage of surface hydroxyl (OH) species. The interaction between OH species and CO intermediates raises the binding energy of CO, thereby facilitating acidic C–C coupling reactions.

Carbon-based materials or supports have demonstrated significant efficacy in enhancing the performance of acidic CO_2_RR. Similar to the neutral or alkaline conditions, catalysts such as Ni SACs,^53,117,118^ Ni_3_N,^117,119^ Ag,^120^ Ni,^121^ and Fe^122^ nanoparticles have shown promising capabilities in generating CO in acidic electrolytes. Importantly, in these studies, the intrinsic structure and properties of carbon materials (*e.g.*, hydrophobicity and porosity) are crucial for ensuring excellent CO_2_RR performance in acidic electrolytes, beyond merely serving as dispersion carriers for single atoms and nanoparticles. For instance, Ma *et al.*^122^ employed a porous carbon layer to encapsulate Fe NPs, creating a local hydrophobic environment that suppressed the HER. Gong *et al.*^123^ embedded Ag into hollow carbon spheres and utilized the confinement effect of carbon to enrich OH^−^, thereby inhibiting the HER. Sargent *et al.* reported a hollow carbon support cobalt phthalocyanine (CoPc/HC) catalyst, where individual CoPc molecules are evenly anchored (Fig. 8e). More importantly, the presence of HC may also help promote the mass transfer of CO_2_ gas and intermediates, although the specific mechanism has not been declared. Notably, Jiang *et al.*^116^ discovered that adding a conductive carbon black layer could maintain a high FE (90%) of CO formation at lower K^+^ concentrations (0.05–0.5 M). They proposed that the carbon black layer protected the silver active sites from directly interacting with carbonates, and thus promoting the accumulation of K^+^ ions and enabling a high FE and stability at lower K^+^ concentrations (Fig. 8f).

## 5. Novel GDE/electrolyzer designs

**Fig. 9:**
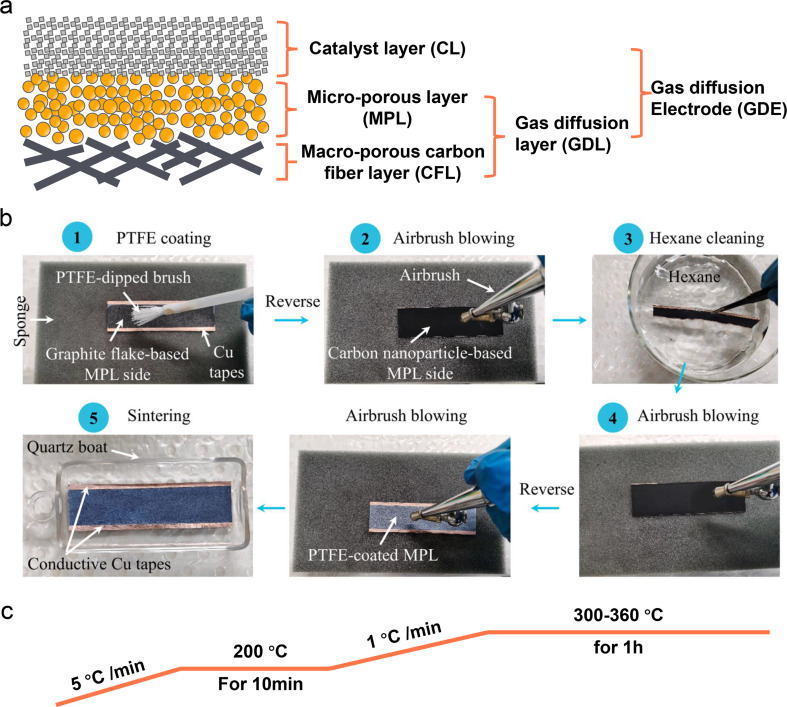
Schematic illustration of a commercial GDL and PTFE-modified GDE. (a) The main components of a typical carbon based GDL and GDE. (b) A hydrophobic modification process of a commercial 28BC (Sigracet) GDE with PTFE emulsion.^124^ (c) A common calcination post-treatment process of a PTFE modified GDE.

Despite some breakthroughs having been achieved in catalyst design or surface modification for the acidic CO_2_RR, the stability, one of the biggest challenges that we have discussed in Section 2, is still unsatisfactory mainly due to the electrolyte flooding of the GDL. The commercial GDL typically includes a macro-porous carbon fiber layer (CFL) and a MPL. Meanwhile, the commonly known GDE is designed by covering a catalyst layer (CL) on the GDL (Fig. 9a). In fact, these commercial GDLs are originally designed for fuel cells, however, the CO_2_RR is much different from those reactions (*e.g.*, HER, ORR) from the aspects of more complex reaction pathways, formation of both gas and liquid products, and so on. Using commercial GDLs to prepare GDEs directly can negatively impact CO_2_RR performance, especially its stability. Therefore, a specialized GDL for the CO_2_RR is critically required. Zhang *et al.*^124^ proposed a hydrophobic modification of a commercial 28BC (Sigracet) GDE (Fig. 9b). They initially used soft nylon bristles to uniformly coat a PTFE emulsion on the MPL surface. To prevent PTFE from blocking the channels in the MPL, they subsequently turned the GDE over and used an airbrush for N_2_ purging. They then cleaned it in hexane and purged it with N_2_ again. After repeating these steps several times, the PTFE-coated 28BC was calcined in a tube furnace under an N_2_ atmosphere (Fig. 9c), resulting in a hydrophobicity-graded GDE. Bao *et al.*^52^ also reported the preparation of a hydrophobic GDL based on commercial carbon paper (Toray TPG-H-60). They mixed carbon black (Vulcan XC-72R) and PTFE in ethanol to form a homogeneous ink, which was then coated directly on the MPL side of the carbon paper. The modified GDL was subsequently calcined in a muffle furnace at 350 °C for 1 h.

**Fig. 10:**
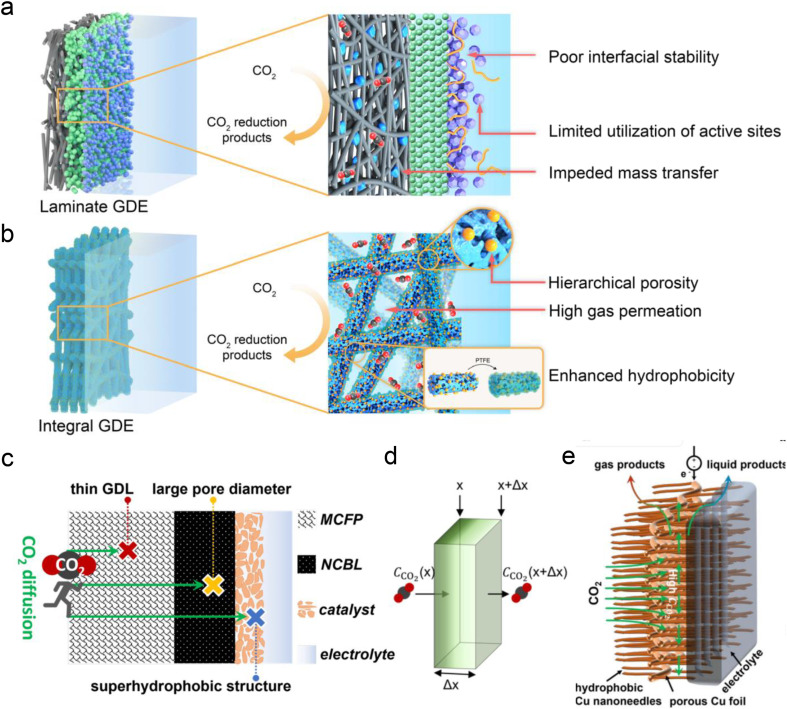
Novel GDE structures for acidic CO_2_RR. (a) Conventional laminate GDE configuration composed of the carbon fiber paper, microporous layer and ionomer-bond catalyst layer.^125^ (b) Novel integral GDE with catalytic sites embedded within the intertwined carbon nanofibers of hierarchical porosity.^125^ (c and d) Illustrations of CO_2_ diffusion in the most traditional GDL and the concentration of CO_2_ decreasing with diffusion distance (Δ*x*). MCFP is microporous carbon fiber paper and NCBL is a nano-microporous carbon black layer.^77^ (e) A novel ultra-thin Cu-based GDL with large pore size and super-hydrophobicity.^77^

Besides the conventional laminate GDE, Peng *et al.*^125^ recently developed a Ni–N–C nanofiber integrated electrode with a graded porous structure using electrospinning technology (Fig. 10a and b). The surface of the electrode was coated with PTFE through heat treatment to enhance its hydrophobicity. This novel GDE exhibits superior mechanical and chemical stability (resistant to water flooding and salting out), more conductive active sites and gas diffusion channels, and simpler fabrication. This novel GDL enables a good stability for CO formation (273 h) in neutral electrolyte with an MEA electrolyzer. However, the FE of CO decreased significantly after 21 h of reaction in acidic electrolyte. This decline may be attributed to salting-out caused by the pH increase at the catalytic interface, thereby affecting the hydrophobic and CO_2_ concentration at the interface, and ultimately intensifying the side reaction of the HER. In addition, to address the issue of CO_2_ diffusion in acidic solutions (Fig. 10c and d), Yamauchi *et al.*^77^ recently proposed a new ultra-thin Cu-based GDL with large pore size and super-hydrophobicity (Fig. 10e). They electrochemically oxidized loose copper sheets under alkaline conditions to form nano-structures with vertical, needle-like surfaces, and then coated them with 1-octadecanethiol to impart waterproof properties. Consequently, the Cu-GDL exhibits a C_2+_ product selectivity of up to 87% in acidic electrolytes and a current density of 1.6 A cm^−2^. Chen *et al.*^30^ proposed the use of copper hollow fibers to enhance electrode penetration. This design compels CO_2_ to interact with active sites as it penetrates the porous wall, thereby improving reaction kinetics at the three-phase interface. The enforced CO_2_ penetration increases its coverage on the electrode surface, effectively suppressing the HER. As a result, they achieved an FE of 70% for C_2+_ products and a single-pass carbon efficiency (SPCE) of 51.8% at a current density of 2 A cm^−2^.

**Fig. 11:**
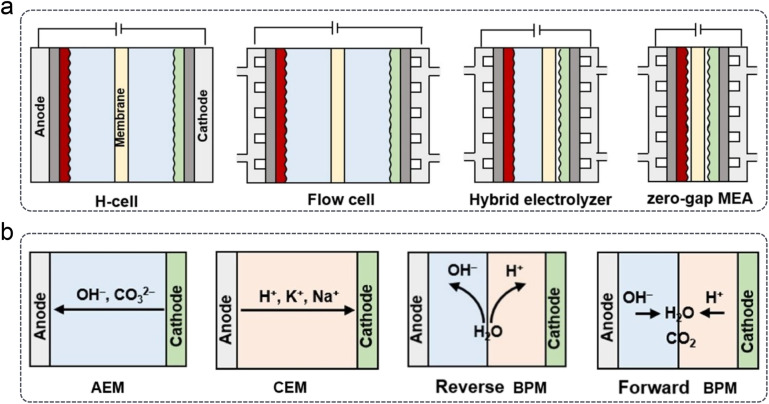
Types of (a) electrochemical cells and (b) polymer electrolyte membrane-based electrolyzers.^36^

In addition to GDE designs, the development of electrochemical cells or electrolyzers is crucial for the industrial application of the CO_2_RR. Based on structural differences, four types of cells can be categorized, as shown in Fig. 11a. Generally, the typical H-cell has been gradually replaced by flow cells or zero-gap MEA electrolyzers, mainly due to their efficient CO_2_ mass transport *via* GDEs. Furthermore, depending on the type of polymer membrane used, electrolyzers can be classified into anion exchange membrane (AEM), CEM, and bipolar membrane (BPM) types, which are commonly employed for the CO_2_RR (Fig. 11b). BPMs can further be divided into reverse and forward types based on the ion movement direction during the water decomposition reaction.

**Fig. 12:**
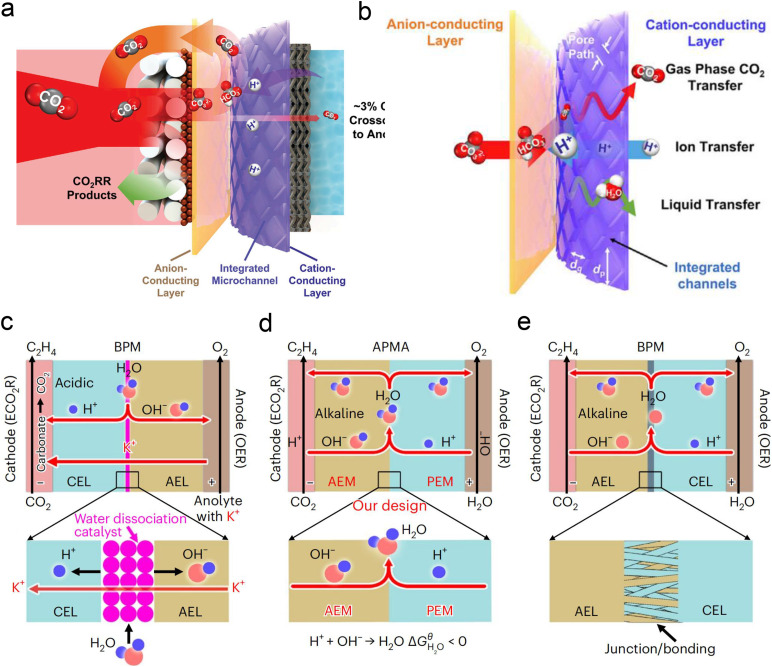
Novel electrolyzer designs. (a) A microchanneled solid electrolyte that internally regenerates and recycles CO_2_, thereby eliminating CO_2_ loss.^126^ (b) Design of parallel flows of microchanneled solid electrolyte for gas-phase CO_2_, liquids, and ions.^126^ (c) The BPM system and reaction with an acidic cathode environment in reverse bias mode.^61^ (d) The APMA system and reaction with an alkaline cathode environment in forward bias mode.^61^ (e) The commercial BPM system and reaction with a bipolar junction/bonding at the anode electrode layer/cathode electrode layer (AEL/CEL) interface in forward bias mode.^61^

Currently, there are limited studies on reactor design for acidic CO_2_RR. Most research still focuses on flow cells or MEA electrolyzers. In flow cells, the use of high-concentration alkali metal cation electrolytes (*e.g.*, 3 M KCl) at the cathode can also leads to the formation of carbonate anions in the local alkaline environment under high current density. These anions react with K^+^ to form (bi)carbonate precipitates, resulting in significant CO_2_ loss in acidic CO_2_RR. Moreover, these hydrophilic precipitates reduce local interface hydrophobicity and may obstruct the flow channels, ultimately decreasing CO_2_RR performance, particularly in terms of selectivity and stability. To address the above problems, Sinton *et al.*^126^ designed a microchannel solid electrolyte (MSE) for acidic CO_2_RR, which can effectively capture and recycle CO_2_ and prevent its loss during electrolysis. The MSE comprises an anion-conducting layer, an integrated channel layer, and a cation-conducting layer near the cathode, resembling the structure of a BPM. Protons migrating from the anode react with carbonate anions from the cathode within the integrated channel layer, regenerating CO_2_ gas molecules that return to the GDE to participate in the reaction (Fig. 12a). The integrated channel layer is fabricated directly on a cation exchange membrane through photolithography, facilitating the transport of gas-phase CO_2_, liquids, and ions *via* parallel flows (Fig. 12b). Additionally, by incorporating a fixed cation in the poly(aryl piperidinium) anion-conducting layer, the quaternary ammonium piperidinium cation can activate CO_2_ reduction in the absence of alkaline cations. The system demonstrated stable operation at a current density of 100 mA cm^−2^ for 200 h in a pure acidic electrolyte (0.01 M H_2_SO_4_), achieving a C_2+_ product FE of 70%. More recently, Lau *et al.*^61^ proposed an AEM + PEM assembly (APMA) MEA system with pure water as the anolyte to avoid the formation of carbonate/precipitation (Fig. 12d). The AEM on the cathode surface provides a local alkaline environment by constraining abundant OH^−^ ions. The presence of PEM allows the transport of H^+^ ions while preventing the crossover of anions from the catholyte. Consequently, only water is formed at the APMA interface. This novel structure differs from conventional bipolar membranes (BPMs) in both reverse (Fig. 12c) and forward bias modes (Fig. 12e). In the reverse bias mode, an additional water dissociation catalyst is required at the interface, while in the forward bias mode, the BPM's stability may be compromised due to difficulties in controlling the mechanical strength of the junction, despite the structural similarity to APMA. Finally, they conducted a scaled-up electrolyzer stack, achieving over 1000 h of stability without CO_2_ or electrolyte losses and a 50% FE for ethylene at a total current of 10 A.

In summary, due to the relatively mature and comprehensive industrial system for water electrolysis and fuel cells, MEA-based electrolyzers are the most promising devices for the industrial application of acidic CO_2_RR.^10^ Despite their relatively complex structure, the above novel APMA electrolyzer serves as an excellent example for long-term electrolysis of pure water CO_2_RR. However, for acidic CO_2_RR in MEA electrolyzers, it remains essential to optimize the operating parameters, such as cell orientation, gas humidification, GDL compression, and cathode CO_2_ pressure.^127,128^ These factors are crucial for ensuring the long-term stability of MEA and the efficiency of the CO_2_RR, forming the foundation for its industrial-scale application.

## 6. Summary and outlook

Since Sargent *et al.*^22^ achieved a milestone breakthrough in acidic CO_2_RR in 2021, significant progress has been made over the past three years. For instance, the FE of C_2+_ products has increased from 30% to nearly 90%, and the current density has reached the A cm^−2^ level. However, acidic CO_2_RR still struggles to maintain good stability at higher current densities, leading to low EE, which falls short of industrial application requirements. This instability may be partly due to the structural degradation of the catalyst itself, as the catalyst is less stable in acidic environments and prone to reconstruction.^18^ Additionally, changes in the microenvironment of the catalytic interface could be a more critical factor affecting stability.^129^ For example, the H^+^/OH^−^ concentration gradient in acidic environments is more pronounced, causing significant local pH changes that impact the stability of the catalyst/electrode interface. Therefore, in this article, we systematically discuss recent strategies for acidic CO_2_RR, focusing on the modification of the electrolyte and local reaction environment as well as the design of GDEs and electrolyzers, to enhance our understanding of the acidic CO_2_RR and meet industrialization requirements in practical applications.

Firstly, electrolyte regulation was one of the earliest and most thoroughly studied aspects, primarily focusing on local pH and cation effects. In traditional neutral or alkaline electrolyte systems, these factors are also crucial and can significantly influence the kinetics of the CO_2_RR. For instance, a high local pH promotes C–C coupling,^68^ a theory that extends to acidic CO_2_RR as well. Koper *et al.*^28^ demonstrated that alkali cations are essential during the CO_2_RR because CO_2_ reduction scarcely occurs without them. Their findings highlighted the key role of alkali cations in stabilizing crucial CO_2_RR intermediates. This theory is equally applicable to acidic CO_2_RR, as the absence of alkali cations in acidic electrolytes results predominantly in H_2_ production. Notably, alkali cations in acidic electrolytes also uniquely repel H^+^ migration. The accumulation of hydrated cations in the OHP inhibits H^+^ transport near the electrode, thereby maintaining the local pH. However, although the effect of alkali cations is crucial for acidic CO_2_RR, it does not ultimately resolve the issue of (bi)carbonate precipitation, as we have discussed in Section 3.2. Interestingly, recent studies have demonstrated that by leveraging the electric field and repulsion effects of cations, coating the catalyst with a layer of organic cations can substitute for alkali metal cations in the electrolyte. This approach enables efficient CO_2_RR in the absence of alkali metal cations and even allows stable operation in pure water electrolyte. These findings suggest that acidic CO_2_RR with low or no metal cations, and even pure water electrolysis, will be a significant research direction in the future.

Secondly, an essential research direction for regulating the local catalytic environment is the surface modification of organic cations, as we have mentioned above. Currently, molecules with organic cationic functional groups mainly include Sustainion, QAPPT, and PDDA. The positive charge density they carry is crucial for their activity. Among them, PDDA exhibits a higher positive charge density and demonstrates superior HER inhibition in pure acid electrolytes.^25^ Therefore, future research should focus on designing functional groups in polymer molecules to enhance positive charge density and maintain a high and stable local hydrogen ion concentration.^130^ On the other hand, current research on the regulation of the local catalytic environment is predominantly based on flow cells, which is mainly concentrated on improving reaction activity and selectivity. Meanwhile, less attention is given to the long-term stability under high current densities, particularly for MEA reactors that offer better commercialization prospects.^10^ Since there is no cathode electrolyte in the MEA electrolyzer, it is more sensitive to potential bicarbonate precipitation caused by the presence of alkali metal cations.^13^ Therefore, achieving higher activity and selectivity in extremely low-concentration or alkali metal cation-free electrolytes is critical for ensuring longer stability in MEA electrolyzers.

Thirdly, regarding GDE/electrolyzer design, current research predominantly relies on porous PTFE or commercial GDLs, both of which have limitations. For instance, PTFE is non-conductive and cannot be directly used as a current collector in an MEA electrolyzer. Additionally, commercial GDLs are typically not designed specifically for the CO_2_RR and are susceptible to electrowetting, leading to interface instability. At present, researchers mainly modify commercial carbon paper with PTFE and carbon black to enhance the hydrophobicity and stability of GDEs. Some have developed new structural GDEs, such as integrated carbon fibers and copper hollow fibers, to improve the selectivity and stability of acidic CO_2_RR by enhancing gas diffusion. However, these GDEs are still only suitable for flow cells. On the other hand, designing a microchannel solid electrolyte layer capable of *in situ* CO_2_ regeneration at the cathode effectively mitigates the carbonate issue.^126^ Additionally, advancements in traditional commercial BPM can address the need for additional hydrolysis catalysts and interface instability, achieving stability for 1000 h in a pure water system.^61^ These findings suggest that for the industrial application of acidic CO_2_RR, the design and development of new GDEs or electrolyzers may be more critical than catalyst research and development.^131^ The underlying scientific challenges and technologies warrant further in-depth investigation.^6^

**Fig. 13:**
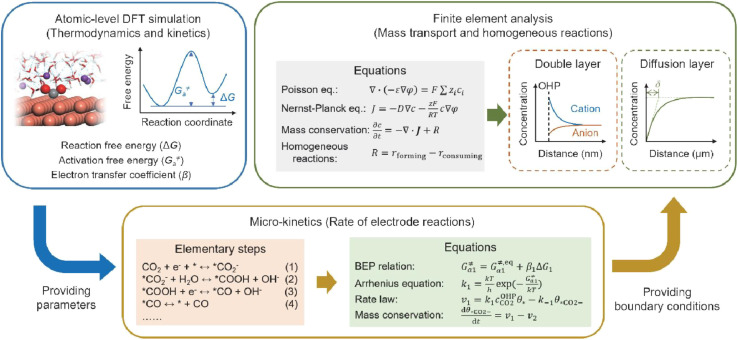
Schematic illustration of simulation methods and process for acidic CO_2_RR.^31^

Fourthly, previous studies have demonstrated that the microenvironment surrounding the cathode—specifically, the local concentration and electric field distribution—significantly influences the performance of the acidic CO_2_RR. However, density functional theory (DFT) simulations are limited in their capacity to elucidate how the CO_2_RR induces alterations in the microenvironment and how these changes subsequently affect the dynamics of the CO_2_RR. To achieve a comprehensive understanding of CO_2_RR performance under acidic conditions, it is crucial to integrate atomic-level DFT simulations with finite element analysis simulations spanning the nanometer to micrometer scale, the latter of which is typically employed to model mass transport and homogeneous reactions. For instance, as illustrated in Fig. 13, DFT-based molecular simulations can first establish the relationship between key intermediates and the local electric field; the energetic parameters obtained can then be utilized to model the micro-kinetics of electrode reactions. Finally, the boundary conditions resulting from electrode reactions can be applied to the finite element analysis of the mass transport process, which includes the EDL, local concentration variations, and the effect of cations or anions.^31^

Finally, SPCE remains a crucial indicator for evaluating the performance of acidic CO_2_RR. A higher SPCE indicates a greater conversion of CO_2_. However, in practical applications, regardless of SPCE, the separation of gas and liquid products from the CO_2_RR presents a technical challenge.^132^ Consequently, it is essential to balance the cost of product separation with the efficiency loss associated with high SPCE, as high SPCE typically results in reduced gas flow rate, current density, and stability, thereby impacting reaction energy efficiency. Resasco *et al.*^132^ recently suggested that maximizing SPCE should not be the primary goal; instead, greater emphasis should be placed on the concentration of the product outlet, which is more critical for practical industrial applications. Apfel *et al.*^78^ also highlighted the substantial gap between fundamental research and industrial application of the CO_2_RR. For instance, the catalyst's overpotential may constitute only a minor portion of the total electrolyzer voltage, and thus it should not be the sole criterion for evaluating GDE performance. They recommended that future research reports provide both half-cell and full-cell data. In summary, acidic CO_2_RR holds significant potential for industrial applications as it can surpass the theoretical 50% conversion limit inherent in traditional neutral or alkaline systems. Despite current technical challenges such as poor selectivity, low energy efficiency, and inadequate stability, it is anticipated that with the ongoing advancement in researchers' understanding of acidic CO_2_RR systems, this technology can achieve practical industrial implementation in future.

## Data availability

Data sharing is not applicable to this article as no new data were generated or analyzed in this study.

## Author contributions

Bangwei Deng and Daming Sun wrote the original draft. Xueyang Zhao, Lili Wang, and Feiyu Ma assisted with the analysis and revised the draft critically. Bangwei Deng, Yizhao Li and Fan Dong reviewed and approved the version of the manuscript to be published.

## Conflicts of interest

There are no conflicts to declare.

## References

[cit1] COP28: What Was Achieved and What Happens Next?, https://unfccc.int/cop28

[cit2] Jouny M., Luc W., Jiao F. (2018). Ind. Eng. Chem. Res..

[cit3] Huang Z., Grim R. G., Schaidle J. A., Tao L. (2021). Energy Environ. Sci..

[cit4] Shin H., Hansen K. U., Jiao F. (2021). Nat. Sustain..

[cit5] Ozden A., De Arquer F. P. G., Huang J. E., Wicks J., Sisler J., Miao R. K., O'Brien C. P., Lee G., Wang X., Ip A. H., Sargent E. H., Sinton D. (2022). Nat. Sustain..

[cit6] Deng B., Huang M., Li K., Zhao X., Geng Q., Chen S., Xie H., Dong X. a., Wang H., Dong F. (2021). Angew. Chem., Int. Ed..

[cit7] Huang M., Deng B. W., Zhao X. L., Zhang Z. Y., Li F., Li K. L., Cui Z. H., Kong L. X., Lu J. M., Dong F., Zhang L. L., Chen P. (2022). ACS Nano.

[cit8] Deng B., Zhao X., Li Y., Huang M., Zhang S., Dong F. (2023). Sci. China Chem..

[cit9] Zhao X., Zhao K., Liu Y., Su Y., Chen S., Yu H., Quan X. (2022). ACS Catal..

[cit10] Ye Q. Q., Zhao X. Y., Jin R. B., Dong F., Xie H. T., Deng B. W. (2023). J. Mater. Chem. A.

[cit11] Bondue C. J., Graf M., Goyal A., Koper M. T. M. (2021). J. Am. Chem. Soc..

[cit12] Gu J., Liu S., Ni W. Y., Ren W. H., Haussener S., Hu X. L. (2022). Nat. Catal..

[cit13] Zhao X., Xie H., Deng B., Wang L., Li Y., Dong F. (2024). Chem. Commun..

[cit14] Kim J., Ha T. H., Kim J., Jeong G. H., Kim S. O., Chung W. S., Roh K., Lee J. H., Oh J. (2023). Appl. Catal., B.

[cit15] Wu Q., Liang J., Han L.-L., Huang Y.-B., Cao R. (2023). Chem. Commun..

[cit16] Zhang J., Guo C., Fang S., Zhao X., Li L., Jiang H., Liu Z., Fan Z., Xu W., Xiao J., Zhong M. (2023). Nat. Commun..

[cit17] Zhang L., Feng J., Liu S., Tan X., Wu L., Jia S., Xu L., Ma X., Song X., Ma J., Sun X., Han B. (2023). Adv. Mater..

[cit18] Löffelholz M., Osiewacz J., Weseler L., Turek T. (2023). J. Electrochem. Soc..

[cit19] Cheng D. F., Alexandrova A. N., Sautet P. (2024). J. Phys. Chem. Lett..

[cit20] Liu H., Yan T., Tan S., Sun L., Zhang Z., Hu S., Li S.-H., Kang X., Lei Y., Jiang L., Hou T., Liu L., Yu Q., Liu B. (2024). J. Am. Chem. Soc..

[cit21] Zi X., Zhou Y., Zhu L., Chen Q., Tan Y., Wang X., Sayed M., Pensa E., Geioushy R. A., Liu K., Fu J., Cortés E., Liu M. (2023). Angew. Chem., Int. Ed..

[cit22] Huang J. E., Li F. W., Ozden A., Rasouli A. S., de Arquer F. P. G., Liu S. J., Zhang S. Z., Luo M. C., Wang X., Lum Y. W., Xu Y., Bertens K., Miao R. K., Dinh C. T., Sinton D., Sargent E. H. (2021). Science.

[cit23] Li L., Liu Z. Y., Yu X. H., Zhong M. (2023). Angew. Chem., Int. Ed..

[cit24] Zhao Y., Hao L., Ozden A., Liu S., Miao R. K., Ou P., Alkayyali T., Zhang S., Ning J., Liang Y., Xu Y., Fan M., Chen Y., Huang J. E., Xie K., Zhang J., O'Brien C. P., Li F., Sargent E. H., Sinton D. (2023). Nat. Synth..

[cit25] Qin H. G., Du Y. F., Bai Y. Y., Li F. Z., Yue X., Wang H., Peng J. Z., Gu J. (2023). Nat. Commun..

[cit26] Yang Y., Shi Y., Yu H., Zeng J., Li K., Li F. (2023). Next Energy.

[cit27] Yin Z. L., Peng H. Q., Wei X., Zhou H., Gong J., Huai M. M., Xiao L., Wang G. W., Lu J. T., Zhuang L. (2019). Energy Environ. Sci..

[cit28] Monteiro M. C. O., Dattila F., Hagedoorn B., García-Muelas R., López N., Koper M. T. M. (2021). Nat. Catal..

[cit29] Deng B. W., Huang M., Zhao X. L., Mou S. Y., Dong F. (2022). ACS Catal..

[cit30] Zhu C., Wu G., Chen A., Feng G., Dong X., Li G., Li S., Song Y., Wei W., Chen W. (2024). Energy Environ. Sci..

[cit31] Zou X. Y., Gu J. (2023). Chin. J. Catal..

[cit32] Hao Q., Liu D. X., Zhong H. X., Tang Q., Yan J. M. (2023). Chem Catal..

[cit33] Xu M., Deng T., Liu L. X., Han X. (2023). Chem.–Eur. J..

[cit34] Yu J. L., Xiao J., Ma Y. B., Zhou J. W., Lu P. Y., Wang K., Yan Y., Zeng J., Wang Y., Song S. Q., Fan Z. X. (2023). Chem Catal..

[cit35] Zhang R. H., Wang H. Y., Ji Y., Jiang Q., Zheng T. T., Xia C. (2023). Sci. China Chem..

[cit36] Zhang T., Zhou J. L., Luo T., Lu J. Q., Li Z. Q., Weng X. X., Yang F. (2023). Chem.–Eur. J..

[cit37] Lee T., Lee Y., Eo J., Nam D.-H. (2024). Nanoscale.

[cit38] Wu W. X., Xu L. P., Lu Q., Sun J. P., Xu Z. Y., Song C. S., Yu J. C., Wang Y. (2024). Adv. Mater..

[cit39] Zeng M., Fang W. S., Cen Y. R., Zhang X. Y., Hu Y. M., Xia B. Y. (2024). Angew. Chem., Int. Ed..

[cit40] Liu Z., Yang H., Kutz R., Masel R. I. (2018). J. Electrochem. Soc..

[cit41] Kim J. Y. T., Zhu P., Chen F.-Y., Wu Z.-Y., Cullen D. A., Wang H. (2022). Nat. Catal..

[cit42] Jeong H.-Y., Balamurugan M., Choutipalli V. S. K., Jeong E.-s., Subramanian V., Sim U., Nam K. T. (2019). J. Mater. Chem. A.

[cit43] Yang H., Kaczur J. J., Sajjad S. D., Masel R. I. (2020). J. CO2 Util..

[cit44] Fan L., Xia C., Zhu P., Lu Y., Wang H. (2020). Nat. Commun..

[cit45] Wu Y., Chen C., Yan X., Wu R., Liu S., Ma J., Zhang J., Liu Z., Xing X., Wu Z., Han B. (2022). Chem. Sci..

[cit46] Xu Y., Li F., Xu A., Edwards J. P., Hung S.-F., Gabardo C. M., O’Brien C. P., Liu S., Wang X., Li Y., Wicks J., Miao R. K., Liu Y., Li J., Huang J. E., Abed J., Wang Y., Sargent E. H., Sinton D. (2021). Nat. Commun..

[cit47] Fang M., Xu L., Zhang H., Zhu Y., Wong W.-Y. (2022). J. Am. Chem. Soc..

[cit48] Zhang L., Li X.-X., Lang Z.-L., Liu Y., Liu J., Yuan L., Lu W.-Y., Xia Y.-S., Dong L.-Z., Yuan D.-Q., Lan Y.-Q. (2021). J. Am. Chem. Soc..

[cit49] Peng C., Xu Z., Luo G., Yan S., Zhang J., Li S., Chen Y., Chang L. Y., Wang Z., Sham T.-K., Zheng G. (2022). Adv. Energy Mater..

[cit50] Li L., Ozden A., Guo S., García de Arquer F. P., Wang C., Zhang M., Zhang J., Jiang H., Wang W., Dong H., Sinton D., Sargent E. H., Zhong M. (2021). Nat. Commun..

[cit51] Xie H., Zhang T., Xie R., Hou Z., Ji X., Pang Y., Chen S., Titirici M.-M., Weng H., Chai G. (2021). Adv. Mater..

[cit52] Li H., Li H., Wei P., Wang Y., Zang Y., Gao D., Wang G., Bao X. (2023). Energy Environ. Sci..

[cit53] Sheng X., Ge W., Jiang H., Li C. (2022). Adv. Mater..

[cit54] Fang W. S., Guo W., Lu R. H., Yan Y., Liu X. K., Wu D., Li F. M., Zhou Y. S., He C. H., Xia C. F., Niu H. T., Wang S. C., Liu Y. W., Mao Y., Zhang C. Y., You B., Pang Y. J., Duan L. L., Yang X., Song F., Zhai T. Y., Wang G. X., Guo X. P., Tan B., Yao T., Wang Z. Y., Xia B. Y. (2024). Nature.

[cit55] Fan M., Miao R. K., Ou P., Xu Y., Lin Z.-Y., Lee T.-J., Hung S.-F., Xie K., Huang J. E., Ni W., Li J., Zhao Y., Ozden A., O’Brien C. P., Chen Y., Xiao Y. C., Liu S., Wicks J., Wang X., Abed J., Shirzadi E., Sargent E. H., Sinton D. (2023). Nat. Commun..

[cit56] Han J., Tu B., An P., Zhang J., Yan Z., Zhang X., Long C., Zhu Y., Yuan Y., Qiu X., Yang Z., Huang X., Yan S., Tang Z. (2024). Adv. Mater..

[cit57] Liu W., Zhai P., Li A., Wei B., Si K., Wei Y., Wang X., Zhu G., Chen Q., Gu X., Zhang R., Zhou W., Gong Y. (2022). Nat. Commun..

[cit58] Kim J.-Y., Hong D., Lee J.-C., Kim H. G., Lee S., Shin S., Kim B., Lee H., Kim M., Oh J., Lee G.-D., Nam D.-H., Joo Y.-C. (2021). Nat. Commun..

[cit59] Wu H., Li J., Qi K., Zhang Y., Petit E., Wang W., Flaud V., Onofrio N., Rebiere B., Huang L., Salameh C., Lajaunie L., Miele P., Voiry D. (2021). Nat. Commun..

[cit60] Li F. W., Thevenon A., Rosas-Hernandez A., Wang Z. Y., Li Y. L., Gabardo C. M., Ozden A., Dinh C. T., Li J., Wang Y. H., Edwards J. P., Xu Y., McCallum C., Tao L. Z., Liang Z. Q., Luo M. C., Wang X., Li H. H., O'Brien C. P., Tan C. S., Nam D. H., Quintero-Bermudez R., Zhuang T. T., Li Y. G. C., Han Z. J., Britt R. D., Sinton D., Agapie T., Peters J. C., Sargent E. H. (2020). Nature.

[cit61] She X., Zhai L., Wang Y., Xiong P., Li M. M.-J., Wu T.-S., Wong M. C., Guo X., Xu Z., Li H., Xu H., Zhu Y., Tsang S. C. E., Lau S. P. (2024). Nat. Energy.

[cit62] Li J., Ozden A., Wan M., Hu Y., Li F., Wang Y., Zamani R. R., Ren D., Wang Z., Xu Y., Nam D.-H., Wicks J., Chen B., Wang X., Luo M., Graetzel M., Che F., Sargent E. H., Sinton D. (2021). Nat. Commun..

[cit63] Fang M., Wang M., Wang Z., Zhang Z., Zhou H., Dai L., Zhu Y., Jiang L. (2023). J. Am. Chem. Soc..

[cit64] Zhong M., Tran K., Min Y., Wang C., Wang Z., Dinh C.-T., De Luna P., Yu Z., Rasouli A. S., Brodersen P., Sun S., Voznyy O., Tan C.-S., Askerka M., Che F., Liu M., Seifitokaldani A., Pang Y., Lo S.-C., Ip A., Ulissi Z., Sargent E. H. (2020). Nature.

[cit65] Zhao Y., Zu X., Chen R., Li X., Jiang Y., Wang Z., Wang S., Wu Y., Sun Y., Xie Y. (2022). J. Am. Chem. Soc..

[cit66] García de Arquer F. P., Dinh C.-T., Ozden A., Wicks J., McCallum C., Kirmani A. R., Nam D.-H., Gabardo C., Seifitokaldani A., Wang X., Li Y. C., Li F., Edwards J., Richter L. J., Thorpe S. J., Sinton D., Sargent E. H. (2020). Science.

[cit67] Adnan M. A., Zeraati A. S., Nabil S. K., Al-Attas T. A., Kannimuthu K., Dinh C. T., Gates I. D., Kibria M. G. (2023). Adv. Energy Mater..

[cit68] Dinh C. T., Burdyny T., Kibria M. G., Seifitokaldani A., Gabardo C. M., de Arquer F. P. G., Kiani A., Edwards J. P., De Luna P., Bushuyev O. S., Zou C. Q., Quintero-Bermudez R., Pang Y. J., Sinton D., Sargent E. H. (2018). Science.

[cit69] Gu Z., Shen H., Chen Z., Yang Y., Yang C., Ji Y., Wang Y., Zhu C., Liu J., Li J., Sham T.-K., Xu X., Zheng G. (2021). Joule.

[cit70] Liu Z., Song L., Lv X., Liu M., Wen Q., Qian L., Wang H., Wang M., Han Q., Zheng G. (2024). J. Am. Chem. Soc..

[cit71] Peng C., Yang S., Luo G., Yan S., Shakouri M., Zhang J., Chen Y., Li W., Wang Z., Sham T.-K., Zheng G. (2022). Adv. Mater..

[cit72] Xu A. N., Hung S. F., Cao A., Wang Z. B., Karmodak N., Huang J. E., Yan Y., Rasouli A. S., Ozden A., Wu F. Y., Lin Z. Y., Tsai H. J., Lee T. J., Li F. W., Luo M. C., Wang Y. H., Wang X., Abed J., Wang Z. Y., Nam D. H., Li Y. C., Ip A. H., Sinton D., Dong C. F., Sargent E. H. (2022). Nat. Catal..

[cit73] Shang L., Lv X., Zhong L., Li S., Zheng G. (2022). Small Methods.

[cit74] Peng C., Yang S., Luo G., Yan S., Shakouri M., Zhang J., Chen Y., Wang Z., Wei W., Sham T.-K., Zheng G. (2023). Small.

[cit75] Chen Y., Li X.-Y., Chen Z., Ozden A., Huang J. E., Ou P., Dong J., Zhang J., Tian C., Lee B.-H., Wang X., Liu S., Qu Q., Wang S., Xu Y., Miao R. K., Zhao Y., Liu Y., Qiu C., Abed J., Liu H., Shin H., Wang D., Li Y., Sinton D., Sargent E. H. (2023). Nat. Nanotechnol..

[cit76] Cao Y. F., Chen Z., Li P. H., Ozden A., Ou P. F., Ni W. Y., Abed J., Shirzadi E., Zhang J. Q., Sinton D., Ge J., Sargent E. H. (2023). Nat. Commun..

[cit77] Sun M., Cheng J., Yamauchi M. (2024). Nat. Commun..

[cit78] Segets D., Andronescu C., Apfel U. P. (2023). Nat. Commun..

[cit79] Neugebauer R. , Wasserstofftechnologien, Springer, Berlin Heidelberg, 2022

[cit80] Bernasconi F., Plainpan N., Mirolo M., Wang Q., Zeng P., Battaglia C., Senocrate A. (2024). ACS Catal..

[cit81] Kalde A. M., Grosseheide M., Brosch S., Pape S. V., Keller R. G., Linkhorst J., Wessling M. (2022). Small.

[cit82] Xie Y., Ou P. F., Wang X., Xu Z. Y., Li Y. C., Wang Z. Y., Huang J. E., Wicks J., McCallum C., Wang N., Wang Y. H., Chen T. X., Lo B. T. W., Sinton D., Yu J. C., Wang Y., Sargent E. H. (2022). Nat. Catal..

[cit83] Liu X., Koper M. T. M. (2024). J. Am. Chem. Soc..

[cit84] Shen J., Kortlever R., Kas R., Birdja Y. Y., Diaz-Morales O., Kwon Y., Ledezma-Yanez I., Schouten K. J. P., Mul G., Koper M. T. M. (2015). Nat. Commun..

[cit85] Nitopi S., Bertheussen E., Scott S. B., Liu X. Y., Engstfeld A. K., Horch S., Seger B., Stephens I. E. L., Chan K., Hahn C., Norskov J. K., Jaramillo T. F., Chorkendorff I. (2019). Chem. Rev..

[cit86] Ooka H., Figueiredo M. C., Koper M. T. M. (2017). Langmuir.

[cit87] Li F.-Z., Qin H.-G., Zhang H.-L., Yue X., Fu L.-K., Xu B., Lin M., Gu J. (2024). Joule.

[cit88] Ling N., Zhang J. G., Wang M., Wang Z., Mi Z. Y., Bin Dolmanan S., Zhang M. S., Wang B. Q., Leow W. R., Zhang J., Lum Y. (2023). Angew. Chem., Int. Ed..

[cit89] Lu X., Zhu C. Q., Wu Z. S., Xuan J., Francisco J. S., Wang H. L. (2020). J. Am. Chem. Soc..

[cit90] Xu Y. N., Li W. J., Fu H. Q., Zhang X. Y., Zhao J. Y., Wu X. F., Yuan H. Y., Zhu M. H., Dai S., Liu P. F., Yang H. G. (2023). Angew. Chem. Int. Ed..

[cit91] Henckel D. A., Counihan M. J., Holmes H. E., Chen X. Y., Nwabara U. O., Verma S., Rodríguez-López J., Kenis P. J. A., Gewirth A. A. (2021). ACS Catal..

[cit92] Zhang J., Guo C. X., Fang S. S., Zhao X. T., Li L., Jiang H. Y., Liu Z. Y., Fan Z. Q., Xu W. G., Xiao J. P., Zhong M. (2023). Nat. Commun..

[cit93] Zhang F., Co A. C. (2019). Angew. Chem., Int. Ed..

[cit94] Wagner A., Sahm C. D., Reisner E. (2020). Nat. Catal..

[cit95] Pan B. B., Wang Y. H., Li Y. G. (2022). Chem Catal..

[cit96] Waegele M. M., Gunathunge C. M., Li J. Y., Li X. (2019). J. Chem. Phys..

[cit97] Frumkin A. N. (1959). Trans. Faraday Soc..

[cit98] Mills J. N., McCrum I. T., Janik M. J. (2014). Phys. Chem. Chem. Phys..

[cit99] Strmcnik D., Kodama K., van der Vliet D., Greeley J., Stamenkovic V. R., Markovic N. M. (2009). Nature Chem..

[cit100] Ringe S., Clark E. L., Resasco J., Walton A., Seger B., Bell A. T., Chan K. (2019). Energy Environ. Sci..

[cit101] Resasco J., Chen L. D., Clark E., Tsai C., Hahn C., Jaramillo T. F., Chan K., Bell A. T. (2017). J. Am. Chem. Soc..

[cit102] Monteiro M. C. O., Philips M. F., Schouten K. J. P., Koper M. T. M. (2021). Nat. Commun..

[cit103] Ma Z. S., Yang Z. L., Lai W. C., Wang Q. Y., Qiao Y., Tao H. L., Lian C., Liu M., Ma C., Pan A. L., Huang H. W. (2022). Nat. Commun..

[cit104] Pan W., Wang P., Fan L., Chen K., Yi L., Huang J., Cai P., Liu X., Chen Q., Wang G., Wen Z. (2023). Inorg. Chem. Front..

[cit105] Pan B., Fan J., Zhang J., Luo Y., Shen C., Wang C., Wang Y., Li Y. (2022). ACS Energy Lett..

[cit106] Singh M. R., Kwon Y., Lum Y., Ager J. W., Bell A. T. (2016). J. Am. Chem. Soc..

[cit107] Ayemoba O., Cuesta A. (2017). ACS Appl. Mater. Interfaces.

[cit108] Qiao Y., Lai W. C., Huang K., Yu T. T., Wang Q. Y., Gao L., Yang Z. L., Ma Z. S., Sun T. L., Liu M., Lian C., Huang H. W. (2022). ACS Catal..

[cit109] Zhang Z.-M., Wang T., Cai Y.-C., Li X.-Y., Ye J.-Y., Zhou Y., Tian N., Zhou Z.-Y., Sun S.-G. (2024). Nat. Catal..

[cit110] Qin H. G., Li F. Z., Du Y. F., Yang L. F., Wang H., Bai Y. Y., Lin M., Gu J. (2022). ACS Catal..

[cit111] Fan M., Huang J. E., Miao R. K., Mao Y., Ou P., Li F., Li X.-Y., Cao Y., Zhang Z., Zhang J., Yan Y., Ozden A., Ni W., Wang Y., Zhao Y., Chen Z., Khatir B., O’Brien C. P., Xu Y., Xiao Y. C., Waterhouse G. I. N., Golovin K., Wang Z., Sargent E. H., Sinton D. (2023). Nat. Catal..

[cit112] Vichou E., Perazio A., Adjez Y., Gomez-Mingot M., Schreiber M. W., Sánchez-Sánchez C. M., Fontecave M. (2023). Chem. Mater..

[cit113] Fan J., Pan B., Wu J., Shao C., Wen Z., Yan Y., Wang Y., Li Y. (2024). Angew. Chem., Int. Ed..

[cit114] Feng S., Wang X., Cheng D., Luo Y., Shen M., Wang J., Zhao W., Fang S., Zheng H., Ji L., Zhang X., Xu W., Liang Y., Sautet P., Zhu J. (2024). Angew. Chem., Int. Ed..

[cit115] Yao Z., Lin R. (2023). Small.

[cit116] Pu Y., Wang Y., Wu G., Wu X., Lu Y., Yu Y., Chu N., He X., Li D., Zeng R. J., Jiang Y. (2024). Environ. Sci. Technol..

[cit117] Zhang J., Lin G., Zhu J., Wang S., Zhou W., Lv X., Li B., Wang J., Lu X., Fu J. (2023). ChemSusChem.

[cit118] Jiang Z., Zhang Z., Li H., Tang Y., Yuan Y., Zao J., Zheng H., Liang Y. (2022). Adv. Energy Mater..

[cit119] Wang Z., Hou P. F., Wang Y. L., Xiang X., Kang P. (2019). ACS Sustain. Chem. Eng..

[cit120] Chen H., Yang K., Shao T., Liu D., Feng H., Chen S., Ortiz-Ledón C. A., Duan J., Li Q. (2023). Electrochim. Acta.

[cit121] Liu Z., Yan T., Shi H., Pan H., Cheng Y., Kang P. (2022). ACS Appl. Mater. Interfaces.

[cit122] Fan Q., Bao G., Chen X., Meng Y., Zhang S., Ma X. (2022). ACS Catal..

[cit123] Li X., Zhang P., Zhang L., Zhang G., Gao H., Pang Z., Yu J., Pei C., Wang T., Gong J. (2023). Chem. Sci..

[cit124] Li L. B., Zhang X. L., Liu C. W., Mosali V. S. S., Chen J., Bond A. M., Gu Q. F., Zhang J. (2023). Appl. Catal., B.

[cit125] Wang M., Lin L., Zheng Z., Jiao Z., Hua W., Wang G., Ke X., Lian Y., Lyu F., Zhong J., Deng Z., Peng Y. (2023). Energy Environ. Sci..

[cit126] Xu Y., Miao R. K., Edwards J. P., Liu S. J., O'Brien C. P., Gabardo C. M., Fan M. Y., Huang J. E., Robb A., Sargent E. H., Sinton D. (2022). Joule.

[cit127] Hoof L., Thissen N., Pellumbi K., junge Puring K., Siegmund D., Mechler A. K., Apfel U.-P. (2022). Cell Rep. Phys. Sci..

[cit128] Oppel N., Röse P., Heuser S., Prokein M., Apfel U.-P., Krewer U. (2024). Electrochim. Acta.

[cit129] Duanmu J. W., Gao F. Y., Gao M. R. (2024). Sci. China Mater..

[cit130] Hall A. S. (2023). Nat. Catal..

[cit131] O’Brien C. P., Miao R. K., Shayesteh Zeraati A., Lee G., Sargent E. H., Sinton D. (2024). Chem. Rev..

[cit132] da Cunha S. C., Resasco J. (2023). Nat. Commun..

